# Placenta and appetite genes *GDF15* and *IGFBP7* are associated with hyperemesis gravidarum

**DOI:** 10.1038/s41467-018-03258-0

**Published:** 2018-03-21

**Authors:** Marlena S. Fejzo, Olga V. Sazonova, J. Fah Sathirapongsasuti, Ingileif B. Hallgrímsdóttir, Vladimir Vacic, Kimber W. MacGibbon, Frederic P. Schoenberg, Nicholas Mancuso, Dennis J. Slamon, Patrick M. Mullin, 23andMe Research Team, Michelle Agee, Babak Alipanahi, Adam Auton, Robert K. Bell, Katarzyna Bryc, Sarah L. Elson, Pierre Fontanillas, Nicholas A. Furlotte, David A. Hinds, Bethann S. Hromatka, Karen E. Huber, Aaron Kleinman, Nadia K. Litterman, Matthew H. McIntyre, Joanna L. Mountain, Elizabeth S. Noblin, Carrie A. M. Northover, Steven J. Pitts, Janie F. Shelton, Suyash Shringarpure, Chao Tian, Joyce Y. Tung, Catherine H. Wilson

**Affiliations:** 1 Division of Hematology-Oncology, David Geffen School of Medicine, Jonsson Comprehensive Cancer Center, University of California at Los Angeles, Los Angeles, CA 90095 USA; 2 Department of Maternal-Fetal Medicine, Keck School of Medicine, University of Southern California, Los Angeles, CA 90033 USA; 3 23andMe, Inc., Mountain View, CA 94041 USA; 4 Hyperemesis Education and Research Foundation, Damascus, OR 97089 USA; 5 Department of Statistics, University of California, Los Angeles, Los Angeles, CA 90095 USA; 6 Department of Pathology and Laboratory Medicine, David Geffen School of Medicine, University of California at Los Angeles, Los Angeles, CA 90095 USA; 7 Present Address: Amgen Inc., South San Francisco, CA 94080 USA

## Abstract

Hyperemesis gravidarum (HG), severe nausea and vomiting of pregnancy, occurs in 0.3–2% of pregnancies and is associated with maternal and fetal morbidity. The cause of HG remains unknown, but familial aggregation and results of twin studies suggest that understanding the genetic contribution is essential for comprehending the disease etiology. Here, we conduct a genome-wide association study (GWAS) for binary (HG) and ordinal (severity of nausea and vomiting) phenotypes of pregnancy complications. Two loci, chr19p13.11 and chr4q12, are genome-wide significant (*p* < 5 × 10^−8^) in both association scans and are replicated in an independent cohort. The genes implicated at these two loci are *GDF15* and *IGFBP7* respectively, both known to be involved in placentation, appetite, and cachexia. While proving the casual roles of *GDF15* and *IGFBP7* in nausea and vomiting of pregnancy requires further study, this GWAS provides insights into the genetic risk factors contributing to the disease.

## Abstract (web summary)

Hyperemesis gravidarum (HG) is a severe form of nausea and vomiting associated with unfavourable outcomes during pregnancy. Here, Fejzo et al. perform genome-wide scans for HG and pregnancy nausea and vomiting and identify genetic associations at two loci implicating the genes *GDF15* and *IGFBP7*.

## Introduction

Nausea and vomiting of pregnancy (NVP) affects 50–90% of pregnant women^1^ and as many as 18% of pregnant women take medication to treat this condition^2^. Hyperemesis gravidarum (HG) is the most severe form and occurs in 0.3–2% of pregnancies^3^. Its clinical presentation includes severe intractable vomiting, often associated with dehydration, weight loss (>5% pre-pregnancy weight), ketonuria, nutritional deficiencies, and electrolyte disturbances^4^. The severity of the disease has been well documented, for instance with the death of author Charlotte Brontë^5^. To this day, HG remains the second leading cause of hospitalization during pregnancy^6^.

Despite the prevalence of NVP and the gravity of HG, decades of research have failed to identify the cause, and a clinically proven, safe, and effective treatment has yet to be found^7^. While the absence of NVP is associated with a higher risk of miscarriage^8^, having the most severe form of nausea and vomiting (HG) is also associated with poor fetal outcomes including preterm birth, neurodevelopmental delay, and vitamin K deficient embryopathy^9–11^. Several possible etiological factors have been investigated but the cause is unknown. Support for a genetic component to NVP come from twin studies in Finnish, Norwegian, and Spanish cohorts^12,13^. Heritability estimates for presence of NVP are as high as 73%^13^. Mothers and sisters of women affected by HG are at increased risk compared to mothers and sisters of unaffected women^14^. The risk of HG is increased by 17-fold for sisters of HG patients, and there is a >27-fold increased risk of mother–daughter recurrence when a mother has HG with two daughters^15,16^.

The aim of this study is to use human genetics as a stepping-stone towards elucidating the causes of HG. Herein, we report a genome-wide association study (GWAS) aimed at identifying variants that influence the risk of HG. We performed two genome-wide association scans, one for a binary HG phenotype, and one for an ordinal phenotype related to HG (severity of NVP). The key findings were replicated in two independent populations of women with clinically defined HG.

## Results

### Genome-wide association scan of the binary phenotype

**Fig. 1:**
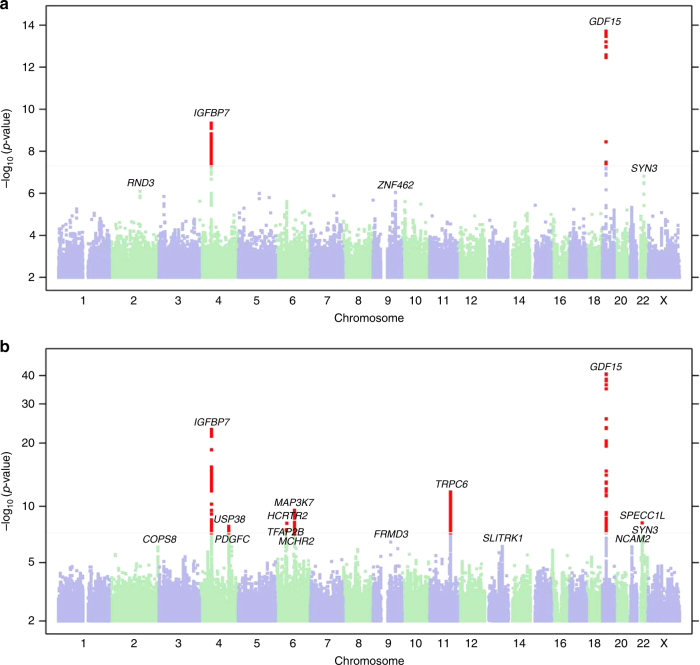
Genome-wide association scans for nausea and vomiting of pregnancy. The Manhattan plot shows distribution of association test statistics vs. genomic position for **a** SCAN1 (binary phenotype), and **b** SCAN2 (ordinal phenotype). The Manhattan plot shows distribution of association test statistics versus genomic position. Chromosomes are arranged along the* X*-axis. Log10-scaled *p*-values are shown on the *Y*-axis. The loci with positions with *p* < 5 × 10^−8^ are shown in red and the loci with *p* < 10^−6^ are labeled with names of nearest genes

For SCAN1, we compared the ends of the clinical spectrum of NVP, HG versus absence of NVP. Participants were female customers of 23andMe, who consented to participate in research, are of European ancestry, and have completed the relevant on-line surveys. A total of 1306 research participants reporting that they received intravenous fluid (IV) therapy for NVP were classified as HG cases, and 15,756 participants who reported no NVP served as controls. Cohort statistics are shown in Table 1. Manhattan and quantile–quantile plots are shown in Fig. 1a and Supplementary Fig. 1a, respectively, and SNP-level QC information is shown in Supplementary Table 1b.

**Table 1:** Demographic characteristics of unrelated female individuals of European descent included in SCAN1

Phenotype	Group	Total	Female	Age < 30	30–44	45–59	≥60
HG	Case	1306	1306	45 (3%)	431 (33%)	555 (42%)	275 (21%)
	Control	15,756	15,756	392 (2%)	3487 (22%)	5357 (34%)	6520 (41%)

Most significantly associated single-nucleotide polymorphisms (SNPs) or insertion or deletion polymorphisms (indels) in each locus are shown in Table 2. Two loci, chr19p13.11 (rs45543339, OR = 0.67, 95% CI [0.60, 0.74], *p* = 1.9 × 10^−14^) and chr4q12 (rs143409503, OR = 0.75 [0.69, 0.82], *p* = 4.5 × 10^−10^), reached genome-wide significance (*p* < 5 × 10^−8^). The 99% credible set for the chr19p13.11 association signal overlapped two genes, *GDF15* and *LRRC25* (see Supplementary Figure 2), and contained a common missense variant in *GDF15* (rs1058587, p.H202D, MAF = 0.25, OR = 0.68 [0.62, 0.75], *p* = 3.4 × 10^–14^) in high linkage disequilibrium (LD) with the lead SNP rs45543339 (*r*^2^ = 0.98, *D*′ = 0.99). The chr4q12 association signal fell within an intergenic region with the closest genes being an uncharacterized lncRNA LOC101928851 (~18.7 kbp away from rs143409503) and protein-coding gene IGFBP7 (~380 kbp away from rs143409503). In addition, there were three more association signals with *p* < 10^−6^ (Table 2).

**Table 2:** Results of the genome-wide association study of no NVP vs HG (SCAN1) with *p* < 10^−6^. The directions of odds ratios (OR) correspond to the minor allele, listed second

Cytoband	Marker	Position	Alleles	MAF	SCAN1 *p*-value	SCAN1 OR	SCAN1 95% CI	SCAN2 *p*-value	SCAN2 effect size	SCAN2 95% CI	Gene context
19p13.11	rs45543339	19:18503194	C/T	0.25	1.9 × 10^−14^	0.668	[0.601,0.743]	5.7 × 10^−39^	−0.104	[−0.120, −0.890]	[LRRC25]
4q12	rs143409503	4:58351064	−/AGC	0.34	4.5 × 10^−10^	0.752	[0.687,0.824]	6.8 × 10^−25^	−0.073	[−0.087, −0.0587]	IGFBP7—[]
22q12.3	rs5998706	22:33440890	C/T	0.49	1.6 × 10^−7^	0.786	[0.717,0.860]	3.5 × 10^−5^	−0.03	[−0.044, −0.016]	[SYN3]
2q23.3	rs114571265	2:150910809	G/C	0.024	8.3 × 10^−7^	0.388	[0.253,0.595]	3.6 × 10^−4^	−0.087	[−0.133, −0.039]	MMADHC—[]—RND3
9q31.2	rs56108151	9:109514426	A/G	0.095	9.2 × 10^−7^	0.677	[0.575,0.797]	5.9 × 10^−4^	−0.034	[−0.062, −0.017]	TMEM38B—[]—ZNF462

### Genome-wide association scan of the ordinal phenotype

For SCAN2, we next conducted a genome-wide association scan of the 53,731 unrelated female 23andMe research participants of European descent, using NVP severity as an ordinal response: none (*N* = 14,988), slight (*N* = 14,292), moderate (*N* = 17,786), severe (*N* = 5445), and very severe NVP (*N* = 1220), coded on a scale of 0 to 4. Details of the definition of each response based on the survey data can be found in the Methods section. Cohort statistics are shown in Table 3. Manhattan and quantile–quantile plots are shown in Fig. 1b, and SNP-level QC information is shown in Supplementary Table 1c.

**Table 3:** Demographic characteristics of unrelated female individuals of European descent included in SCAN2

Phenotype	Group	Total	Age < 30	30–44	45–59	≥60
NVP	None	14,988	373 (2.5%)	3332 (22.2%)	5129 (34.2%)	6154 (41.1%)
	Slight	14,292	233 (1.6%)	2635 (18.4%)	4831 (33.8%)	6593 (46.1%)
	Moderate	17,786	391 (2.2%)	3692 (20.8%)	6233 (35.0%)	7470 (42.0%)
	Severe	5445	140 (2.6%)	1298 (23.8%)	1580 (29.0%)	2427 (44.6%)
	Very severe	1220	42 (3.4%)	409 (33.5%)	519 (42.5%)	250 (20.5%)

The two most strongly associated loci in SCAN2 were also genome-wide significant in SCAN1: chr19p13.11 (rs16982345, *β* = −0.104 [−0.12, −0.089], *p* = 2.4 × 10^−41^) and chr4q12 (rs143409503, *β* = −0.072 [−0.086, −0.058], *p* = 9.2 × 10^−24^). Five additional regions reached genome-wide significance in SCAN2 (Table 4; Supplementary Figure 3) and there were eight additional association signals in SCAN2 with *p* < 10^−6^ (Supplementary Table 2).

**Table 4:** Results of GWAS of NVP as an ordinal response (SCAN2) with *p* < 5 × 10^−8^. The directions of effect correspond to the minor allele, listed second

Cytoband	Marker	Position	Alleles	MAF	*p*-value	*β*	95% CI	SCAN1 *p*-value	SCAN1 OR	SCAN1 95% CI	Gene context
19p13.11	rs16982345	19:18500722	G/A	0.25	2.4 × 10^−41^	−0.104	[−0.120, −0.089]	6.81 × 10^−15^	0.68	[0.615, 0.751]	GDF15[]-LRRC25
4q12	rs143409503	4:58351064	−/AGC	0.34	9.2 × 10^−24^	−0.072	[−0.086,−0.058]	1.86 × 10^−10^	0.752	[0.688, 0.822]	IGFBP7—[]
11q22.1	rs2508362	11:101260798	A/G	0.199	1.7 × 10^−12^	0.06	[0.043,0.076]	1.06 × 10^−04^	0.819	[0.741, 0.904]	PGR—[]–TRPC6
6q15	rs4707680	6:92294371	T/C	0.427	2.8 × 10^−10^	0.043	[0.030, 0.056]	5.79 × 10^−02^	0.924	[0.851, 1.00]	MAP3K7-[]
22q11.23	rs201838815	22:24753023	T/−	0.02	5.8 × 10^−9^	−0.159	[−0.212, −0.105]	2.88 × 10^−02^	0.685	[0.480, 0.979]	[SPECC1L,SPECCl-ADORA2A]
6p12.1	rs7761177	6:55151343	T/C	0.495	6.2 × 10^−9^	−0.039	[−0.052, −0.026]	1.70 × 10^−06^	0.82	[0.755, 0.889]	HCRTR2-[]–GFRAL
4q31.21	rs4690766	4:144030040	G/C	0.358	1.3 × 10^−8^	−0.04	[−0.054, −0.026]	1.02 × 10^−01^	0.932	[0.856, 1.01]	INPP4B—[]–USP38

### Replication

Two loci were genome-wide significant in both association scans and were selected for replication genotyping. Given that the replication cohorts contain information on HG as a binary trait and that HG is the more clinically relevant phenotype, three additional loci with *p* < 10^−6^ in the binary trait scan (SCAN1) were also selected for replication. The first replication cohort (HG-IV) included 789 women with HG requiring IV fluid treatment and 606 controls reporting normal NVP. The second replication cohort (HG-TPN) included only women at the extreme ends of the clinical spectrum, 110 women requiring total parenteral nutrition (TPN) and 143 women reporting no NVP. Basic demographics of the replication cohorts are shown in Table 5. Over 90% of participants self-reported that they were of European descent.

**Table 5:** Demographic characteristics of the replication cohorts HG-IV consisting of HG patients treated with intravenous (IV) fluids and controls with untreated NVP, and HG-TPN consisting of HG patients treated with total parenteral nutrition (TPN) and controls with no NVP

	Total	Year born (average)	Ethnicity (European descent)	Attended college	Advanced degree	First child year born
*HG-IV*						
HG	789	1977	90%	61%	19%	2003
Control	606	1975	92%	62%	18%	2002
*HG-TPN*						
HG	110	1976	90%	61%	26%	2002
Control	143	1974	93%	51%	25%	2001

We selected rs16982345 in the chr19p13.11 locus as a proxy for the lead SNP rs45543339 (*r*^2^ = 0.98, *D*′ = 0.99) for replication in HG-IV (because rs16982345 had a commercially available high quality SNP assay using the Taqman genotyping system). rs16982345 was successfully genotyped in 789 individuals with clinically defined HG and 581 controls reporting normal NVP using the TaqMan genotyping platform (Table 6). The call rate was >95%. The replication results for rs16982345 were supportive of the GWAS result (*p* = 2.8 × 10^−7^, OR = 1.63 [1.35, 1.98]).

**Table 6:** Replication results based on Fisher’s exact test for the two most significantly associated loci in SCAN1 and SCAN2: rs16982345 (chr19p13.11), rs4865234 (chr4q12), and the three association signals in SCAN1 with *p* > 5 × 10^−8^ and *p* < 1 × 10^−6^, rs5754397 (SYN3) rs78353059 (MMADHC/RND3), and rs56108151 (TMEM38B/ZNF462)

	*N*	Genotype 1	Genotype 2	Genotype 3	*p*-value	OR	95% CI
*rs16982345*		GG	AG	AA			
HG-IV	789	540	229	20			
CONTROL	581	330	210	41	2.80 × 10^−7^	1.63	1.35–1.98
HG-TPN	103	68	33	2			
NO NVP	136	75	51	10	0.04	1.61	1.01–2.60
*rs4865234*		AA	AG	GG			
HG-IV	778	404	312	62			
CONTROL	603	259	273	71	3.50 × 10^−4^	1.35	1.14–1.59
HG-TPN	110	64	38	8			
NO NVP	143	57	66	20	2.81 × 10^−3^	1.81	1.21–2.73
*rs5754397*		CC	CT	TT			
HG-IV	774	218	353	203			
CONTROL	606	180	273	153	0.51	0.95	0.82–1.11
HG-TPN	106	37	44	25			
NO NVP	141	44	64	33	0.72	1.07	0.74–1.56
*rs78353059*		AA	AG	GG			
HG-IV	779	735	42	2			
CONTROL	596	561	34	1	0.82	1.07	0.66–1.71
HG-TPN	105	99	6	0			
NO NVP	136	125	11	0	0.62	1.43	0.48–4.80
*rs56108151*		AA	AG	GG			
HG-IV	780	625	150	5			
CONTROL	594	495	97	2	0.06	0.78	0.59–1.02
HG-TPN	108	86	22	0			
NO NVP	141	117	23	1	0.64	0.86	0.45–1.65

The number (*N*) for each group is the total number of samples that were successfully genotyped. The call rate was >95%. OR estimates, 95% confidence intervals, and *p*-values were computed using 2 × 2 contingency tables in R. The effect allele is assumed to be the allele in the left-most (i.e., first) homozygous cell

We also successfully genotyped the variant in 103 women with HG who required TPN and 136 women who reported no NVP in at least two pregnancies (HG-TPN). The genotyping results had comparable effect size, but were not significant after multiple testing correction (*p* = 0.04, OR = 1.61 [1.01, 2.60]) (Table 6).

We also performed replication of the second genome-wide significant locus. rs4865234 was used as a proxy for rs143409503 (*r*^2^ = 0.95, *D*′ = 1.0) (because rs143409503 is an indel, which cannot be assayed using the TaqMan genotyping system). rs4865234 replicated in HG-IV (*p* = 3.5 × 10^−4^, OR = 1.35 [1.14, 1.59]) (Table 6). The genotyping results of DNA from HG-TPN were also supportive (*p* = 2.8 × 10^−3^, OR = 1.81 [1.21, 2.73]).

The three additional SNPs with *p* < 10^−6^ in the binary trait scan (rs5754397, rs78353059, rs56108151) did not replicate in HG-IV nor in HG-TPN (*p* > 0.05, see Table 6).

### Combined analysis

For the replicated loci, we combined the replication results with SCAN1 using a fixed-effect meta-analysis (Table 7). Briefly, this test computes a weighted average of odds-ratio estimates across studies while accounting for standard errors. For rs16982345 on chr19p13.11, we estimated a meta-OR of 1.50 [1.38, 1.65] (*p* = 2.12 × 10^−19^). Heterogeneity of individual effect sizes did not appear to influence the combined estimate (*p*_het_ = 0.66). For rs4865234 on chr4q12, the meta-OR was 1.33 [1.23, 1.62] (*p* = 9.29 × 10^−12^) with little evidence of effect size heterogeneity (*p*_het_ = 0.727).

**Table 7:** Results of meta-analysis of SCAN1 and replication cohorts

SNP	CHR	BP	A0	A1	Study	HG	CONTROL	OR	95% CI	*p-value*	Het *p*
						A1 Freq	A1 Freq				
rs16982345	chr19	18500722	A	G	SCAN1	0.8	0.73	1.47	[1.33, 1.63]	6.81E−15	–
					HG-IV	0.83	0.75	1.63	[1.35, 1.98]	2.80E−07	–
					HG-TPN	0.82	0.74	1.61	[1.01, 2.60]	4.00E−02	–
					META	–	–	1.5	[1.38, 1.65]	2.12E−19	6.62E−01
rs4865234	chr4	58355015	G	A	SCAN1	0.7	0.64	1.32	[1.20, 1.43]	7.95E−10	–
					HG-IV	0.72	0.66	1.43	[1.16, 1.79]	9.00E−04	–
					HG-TPN	0.75	0.63	1.82	[1.20, 2.70]	2.81E−03	–
					META	–	–	1.33	[1.23, 1.62]	9.29E−12	7.27E−01

A1 is the allele associated with increased risk of HG

### Conditional analyses

In order to identify additional association signals, we performed stepwise conditional analysis at all loci that reached genome-wide significance in either SCAN1 or SCAN2. Conditional analysis detected secondary associations in the chr19p13.11 locus in SCAN1 and in the chr19p13.11 and *PGR*/*TRPC6* loci in SCAN2 (Supplementary Table 3 and Supplementary Figures 4 and 5). LD between the primary and secondary signals is low (*r*^2^ < 0.01), suggesting that they are independent signals corresponding to multiple risk variants within the locus.

The secondary signal in the chr19p13.11 locus identified from SCAN1 (rs1054221, OR = 1.38 [1.22, 1.56], *p* = 1.7 × 10^−7^) maps within the 3′ UTR of *GDF15*. The secondary signal from SCAN2 in this same locus is a common, single-nucleotide deletion with an effect comparable to the primary association (rs34345957, *β* = 0.11 [0.089, 0.13], *p* = 1.1 × 10^−28^, MAF = 0.14), located in the intron of *LRRC25*, and in high LD (*r*^2^ = 0.98, *D*′ = 1.0) with the secondary signal in SCAN1. The tertiary association signal within the chr19p13.11 locus in SCAN2 is weaker (rs4808787, *β* = −0.037 [−0.053, −0.021], *p* = 6.0 × 10^−6^) and is located in the 5′ UTR of the long isoform of *PGPEP1*.

### Functional analyses of chr19p13.11 and chr4q12 genomic loci

The genomic context of the replicated loci chr19p13.11 and chr4q12 was analyzed using HaploReg^17^ (Supplementary Data 1). Within those two regions, we analyzed A) the index SNPs for strongest association in SCAN1 (rs45543339 and rs143409503) and the proxy SNPs that were confirmed in B) the independent replication cohort (rs16982345 and rs4865234).

The lead SNP at chr19p13.11, rs45543339, was located in an intron of *LRRC25*, and the only notable functional annotation was an eQTL for *KCNN1* in mucosa of the esophagus. rs16982345, which is within the 99% credible set at this locus and is in tight LD (*r*^2^ = 0.98) with the lead SNP rs45543339, is located within an intron of *GDF15* (Supplementary Data 1 and 2). It overlapped enhancer histone marks in three tissues, including placenta, and DNAse hypersensitivity sites in nine tissues, including ovary. A summary of the variants in LD with the lead SNP and associated with altered *GDF15* expression is shown in Supplementary Data 2^17–20,39–42,44^, with the full list of variants in LD with the lead SNP identified using HaploReg listed in Supplementary Data 1. One of the variants associated with HG in SCAN1, rs17725099 (*p* = 1.03 × 10^−13^) and in LD with rs16982345 (*r*^2^ = 0.77), was the top association signal (*β* = 0.16, *p* = 1.47 × 10^–107^) in a conference abstract which identified variants influencing *GDF15* levels in patients with cardiovascular disease^18^. The SNP rs17725099 was also associated with circulating levels of GDF15 in the first study to report on the heritability of GDF15 plasma concentration^19^. In addition, rs1058587, a SNP in perfect LD (*r*^2^ = 1) with the lead SNP and a part of the 99% credible set at this locus in SCAN1, has previously been associated with altered GDF15 expression. Expression levels of *GDF15* were compared in an in vitro model between the cell line DU145 transfected with wild-type *GDF15* (rs1058587, C allele) and *GDF15* (rs1058587, G allele)^20^. The C allele of rs1058587 was associated with increased GDF15 levels in the in vitro model and was also the risk allele associated with HG, providing evidence that the direction of effect is toward higher levels of GDF15 for HG compared to controls.

The lead indel at chr4q12, rs143409503, mapped to an intergenic region with the closest protein-coding gene being *IGFBP7*, ~380 kbp away from the marker. No notable features were identified using HaploReg^17^, the GTEx portal, nor ExSNP^21^ databases. Other phenotypes associated with SNPs in *GDF15* and *IGFBP7* are reported in Supplementary Note 1^41–56^.

## Discussion

This GWAS of HG identified two genome-wide significant signals that were subsequently replicated in the larger of two independent cohorts (HG-IV). The most significantly associated locus on chr19p13.11 contained genes *GDF15* and *LRRC25*. While we cannot be certain which gene or genes are implicated, SNPs in LD with the associated variants at the chr19p13.11 locus were found to be associated with altered expression of *GDF15*. In addition, the gene encoding the receptor for GDF15, the brainstem-restricted receptor GFRAL^22^, was associated with NVP in SCAN2 (Table 4) adding further evidence supporting a previously unknown biological connection between *GDF15* and HG. While the results of this study do not establish a causal link between *GDF15* and HG, the association between this gene and HG is of particular importance because it highlights the possibility of a pathway involved in the etiology of the condition. *GDF15* encodes a TGF-β superfamily member that is expressed at its highest levels in the trophoblast cells of the placenta^23^. The protein is found in maternal serum and increases significantly in the first two trimesters^23^. GDF15 is believed to suppress production of proinflammatory cytokines in order to facilitate placentation and maintain pregnancy^24^. In addition to its role in pregnancy, GDF15 has been shown to be a regulator of physiological body weight and appetite via activation of neurons in the hypothalamus and area postrema (vomiting center) of the brainstem^25,26^. It is also notable that abnormal overproduction of GDF15 in cancer was recently found to be the key driver of cancer anorexia and cachexia which, like HG, exhibits symptoms of chronic nausea and weight loss^27,28^. Of particular clinical interest, inhibition of GDF15 restored appetite and weight gain in a mouse model of cancer cachexia^28^, suggesting a therapeutic strategy that may be applicable to patients with HG, if *GDF15* proves to be the implicated gene.

The other locus that reached genome-wide significance and was confirmed in the independent replication cohort, is chr4q12. Although there were no genes that can be directly related to the association signal, the protein-coding gene closest to the lead variant is *IGFBP7*. We have not found eQTLs that link the variants associated in this study to expression of *IGFBP7*, however, this can be explained at least in part by a lack of eQTL data in relevant tissues during pregnancy. The notably similar and functionally relevant roles of *IGFBP7* and *GDF15* (in placentation, cachexia, and feeding behavior) make a compelling argument for *IGFBP7* despite the lack of evidence linking the association signal to its expression. Insulin-like growth factor binding protein 7 (*IGFBP7*) is a gene involved in implantation and decidualization of the pregnant uterus^29^. Like *GDF15*, *IGFBP7* is significantly upregulated after implantation and highly expressed in the developing placenta^30^. Inhibition of *IGFBP7* caused pregnancy loss in a mouse model by shifting uterine cytokines to Th1 type dominance and repressing uterine decidualization^30^. Thus *IGFBP7* may play roles in both miscarriage and in the severity of NVP, providing a genetic mechanism for the protective effect of NVP. In addition, like GDF15, evidence points to IGFBP7 as a promising biomarker for cachexia associated with end-stage disease^29^. An intriguing finding is that the *Drosophila* homolog of *IGFBP7* has been suggested to play a role in neuronal coordination between metabolic status and feeding behavior, potentially, like with HG, causing food aversion to normally attractive food, even when starving, and vice versa^31^.

The strengths of this study include the well-defined phenotype and large sample sizes in two scans and the HG-IV replication sample. The strong independently replicated association signals, and the fact that both candidate genes have roles in early pregnancy when HG sets in, support these genes as functional as well as positional candidate genes^30,32^. However, it is important to replicate the findings reported herein, both using larger replication sample sizes (for HG-TPN vs no NVP) and in studies of different ethnicities to determine whether the findings can be validated and generalized to other populations. Another caveat is that while the conditional analysis yielded additional association signals at chr19p13.11 that may act independently of the lead association signal, they were not replicated. Replication is necessary to determine if additional independent variants are associated at this locus. Finally, the lack of eQTL data and considerable distance between the chr4q12 association signal and the closest gene *IGFBP7* is a noteworthy limitation.

Future research should focus on functional follow-up. Since GDF15 and IGFBP7 levels are upregulated during placentation and cachexia^23,24,28,29^, and downregulated prior to miscarriage^30,32^, serum concentrations of GDF15 and IGFBP7 should now be studied in pregnant women with and without HG. If GDF15 or IGFBP7 prove to be relevant, it is conceivable that drugs targeting these proteins may have clinical utility in treating HG. Such a study may also lead to new methods for prediction and diagnosis. In addition, the SCAN2 results suggest a great potential for discovering additional loci associated with HG or NVP. Finally, the findings herein suggest an answer to an age-old paradox. HG can lead to prolonged dehydration and undernutrition, which can be detrimental to maternal and fetal health and can decrease reproductive fitness. The dual roles of GDF15 and IGFBP7 in maintaining pregnancy and in increasing the risk of HG may provide a molecular explanation for why NVP still exists in nature.

## Methods

### Human subjects for GWAS

GWAS participants were customers of 23andMe, Inc. who consented to participate in research, and provided answers to the morning sickness-related questions. Two genome-wide association scans were performed, for (1) a binary HG phenotype and (2) an ordinal phenotype related to HG. Phenotype definitions are described below. Due to the nature of the phenotype definitions, some research participants were included in both association scans. All research participants included in the analysis provided informed consent and answered on-line surveys according to a human subjects protocol approved by Ethical & Independent Review Services, a private institutional review board.

### Phenotype definitions

For the binary HG phenotype analyzed in SCAN1, we compared two ends of the clinical spectrum of NVP: 1306 research participants who reported via an on-line survey that they received IV therapy for NVP were classified as HG cases and 15,756 participants who reported no NVP served as controls. The phenotype definition was ascertained using the interview questions listed in Supplementary Note 2. For the ordinal phenotype related to HG analyzed in SCAN2, data were pulled from four questions also listed in Supplementary Note 2.

### Genotyping and imputation and association testing

A total of 17,062 females in SCAN1 and 53,731 females in SCAN2 were genotyped on one of four custom Illumina genotyping arrays and additional genotypes were imputed using the September 2013 release of the 1000 Genomes Project Phase 1^33^ reference haplotypes as described previously^34,35^. 16,165 individuals in SCAN1 were also included in SCAN2. Breakdown of the number of participants in specific phenotype categories in SCAN1 and SCAN2 are shown in Supplementary Table 1a. Fields that contain 5 or fewer individuals have been masked to protect the privacy of 23andMe customers. All females were filtered to select for European ancestry, and close relatives were removed. We performed logistic (SCAN1) and linear (SCAN2) regression assuming an additive model for allelic effects on NVP, using age and five principal components of genetic ancestry as covariates. Our previously published analysis of categorical phenotypes using ordinal and linear regression showed high concordance between resulting *p*-values^36^. Due to comparative ease of implementation and lower computational burden, we used linear regression in SCAN2.The SNP-level quality control information is shown in Supplementary Tables 1b and 1c. Test statistics were further adjusted for the genomic control inflation factor of *λ* = 1.044 for SCAN1 and *λ* = 1.087 for SCAN2. The index SNPs were identified by choosing the SNPs with the strongest association in each associated region. Each region contained SNPs with *p* < 10^−5^ that were grouped into intervals separated by a gap of a minimum of 250 kbp. The SNP with the smallest *p*-value within each interval was chosen as the lead SNP.

### Credible sets

To account for the uncertainty in identifying the true causal variant within an association signal, we computed 99% credible sets at each locus. Under the assumptions that there is a single causal variant at a locus and that this variant was genotyped in the association study, 99% credible sets are defined as smallest sets of variants that are 99% likely, based on posterior probability, to contain the causal variant^37^. While these assumptions are not always true in practice, credible sets provide a helpful summary of the available evidence that one of the SNPs contained in them is causal, and may help in fine-mapping the association signal.

### Replication cohorts

The first replication cohort (HG-IV) included 789 HG cases treated with IVs and 606 controls with NVP that did not require treatment. The second replication cohort (HG-TPN) included 110 cases requiring TPN due to HG and 143 controls reporting no NVP in any of at least two pregnancies. All participants gave informed consent. This study was approved by the UCLA Institutional Review Board.

### Phenotype definition in the replication cohort

Affected individuals were included if they had an HG diagnosis and were treated with IV fluids and/or TPN. Participants affected by HG recruited acquaintances that were non-blood related and had at least two pregnancies lasting more than 27 weeks gestation. Controls were eligible to participate in the study if they reported normal or no NVP, did not lose weight due to nausea/vomiting, and did not receive medical attention due to NVP.

### Recruitment of the replication cohort

For the replication study, the source population for HG cases included patients residing in the US. The majority of cases were recruited through a posting from 2007 to 2017 on the Hyperemesis Education and Research Foundation website (www.HelpHer.org). Minors (under 18 years) were excluded because the risk to benefit ratio to control minors would be difficult to justify and the study requires controls to have had two pregnancies, which is uncommon in minors. Women with pregnancies with chromosome abnormalities and multiple gestations were excluded due to possible distinct etiologies for NVP in these cases. Medical records and recruitment of an acquaintance with at least two pregnancies to serve as a control, were requested of each case. All participants were required to go over an information sheet by phone and return a signed information sheet with all elements of consent in order to enroll in the study.

### Sample collection

Saliva samples were collected for DNA analysis from all cases and controls. DNA Genotek saliva kits (Oragene, Ottawa, Canada) were mailed to all cases and controls. The saliva collection kit is self-administered and comes with directions for submitting 2 ml of saliva into a collection vial and returning the sample to the study site via an addressed and postage-paid return envelope provided with the collection kit.

### DNA extraction

DNA was extracted from the saliva samples according to manufacturer’s instructions (Oragene, Ottawa Canada). Using the kit, we have successfully isolated, on average, 197 µg of DNA of high quality (260/280 1.84) from 2 ml of saliva. The low end of expected DNA quantity reported by the manufacturer is 30 µg/ml of saliva or 60 µg/sample. After the extraction, the DNA was stored at −20 °C.

### Quality control for replication data

The TaqMan genotyping platform was performed on 384-well plates with a minimum of two blank samples per plate and a minimum of two duplicate samples per plate. Once genotypes were determined from the first 384-well plate, a minimum of three positive controls or one positive control for each genotype was added to the remaining plates. The minimum call rate for each SNP was >95%.

### Genotyping and statistical analysis in the replication study

rs16982345, the SNP with the strongest association to NVP in the chr19p13.11 locus was selected for genotyping in the replication sample HG-IV consisting of 789 cases and 606 controls. For the non-coding marker rs16982345 in chr19p13.11, genotyping a minimum of 780 cases and 580 controls, and assuming a proportion of the GG genotype similar to SCAN1 (0.53 for controls and 0.64 for cases), we estimated a power greater than 97% to detect between-group genotype differences using a two-sample, two-sided test of proportions at significance level alpha = 0.05.

In addition, we genotyped an extreme cohort (HG-TPN) consisting of 110 women who reported TPN for HG and 143 controls with no NVP in two or more pregnancies. TaqMan genotyping primers for rs16982345 were available from Thermo Fisher Scientific and Applied Biosystems PRISM 7900HT Sequence Detection System (TaqMan) was used for large-scale screening. The call rate was >95%.

Genotypes were tested for significant association with HG using Fisher’s exact test using 2 × 2 contingency tables counting the number effect vs non-effect alleles for cases and controls using R.

The SNP rs4865234 was genotyped as a proxy for the most significant association signal at chr4q12 because the most significant association signal, rs143409503, is an indel, which cannot be assayed using the TaqMan genotyping system. The SNP rs4865234 is in tight LD with rs143409503 (*r*^2^ = 0.95, *D*′ = 1.0) and genotyped on DNA isolated from HG-IV and HG-TPN cohorts as stated previously for rs16982345.

The 3 additional loci that reached significance *p* < 10^−6^ in the binary trait scan (SCAN1) were also genotyped in the two replication cohorts (HG-IV and HG-TPN) using the TaqMan genotyping platform. The SNP rs5754397 was used as a proxy for the lead SNP rs5998706 (*r*^2^ = 0.55, *D*′ = 0.83), and the SNP rs78353059 was used as a proxy for the lead SNP rs114571265 (*r*^2^ = 0.87, *D*′ = 1.0) because they were the closest SNPs listed in HaploReg^17^ to the lead SNP with an available commercially validated assay from Thermo Fisher Scientific and they also reached significance *p* < 10^−6^ in SCAN1. The lead SNP rs56108151 was commercially available and used for genotyping the two replication cohorts.

### Meta-analysis of SCAN1 and replication

We performed a fixed-effect meta-analysis using the R language and software *meta* to compute the weighted average odds-ratio across the replication group and SCAN1 for sites rs16982345 and rs4865234. The fixed-effect meta-analysis assumes that there exists a true underlying effect in the population and combines individual study estimates (e.g., replication and SCAN1) to obtain a meta estimate. This assumption may be invalid in practice due to differences in linkage disequilibrium patterns across studies, or differences in covariates included. To test for hetereogeneity in effects across studies, or significant study-wide variance, we estimated Cochran’s Q^38^ for each SNP. This measures the variance across studies using the meta estimate as the true mean, which can be tested in a null-hypothesis framework under a $\chi _{n - 1}^2$ distribution. Here, the number of studies is $n = 2$, so our heterogeneity *p*-values reflect a one-tailed test under a $\chi _1^2$ distribution.

### Conditional analysis

The stepwise conditional analysis detects statistically independent signals in a GWAS locus. At each step, we assessed association between variants within 20 kbp around a GWAS locus and the HG or related ordinal phenotype, using top SNPs from preceding steps as additional covariates. This was repeated until no significant association was detected at *p* < 10^−5^. Finally, we fit a joint model with all of the primary and secondary signals and evaluated its goodness of fit through the likelihood ratio test (LRT) against the model with just the primary signal. The reported effect sizes of the secondary signals are from the joint model, and the *p*-values are from the likelihood ratio test comparing the full model with the leave-one-out models.

### In silico functional follow-up

We used the HaploReg^17^ v4.1 tool to identify SNPs in the haplotype blocks containing the two significant GWAS association signals, their LD *r*^2^ and *D*′ scores, associated alleles, allele frequencies, genes, functional annotation, conservation, regulatory elements, protein-binding sites, motif changes, and eQTLs. We queried the two most significant loci identified, chr19p13.11 and chr4q12. Within those two regions, we analyzed SNPs with HaploReg, which uses LD information from the 1000 Genomes Project^33^. The LD threshold in HaploReg v4.1 was set at *r*^2^ = 0.2: the population used for LD calculations was the 1000 Genomes Project Phase 1 European descent: the source for epigenomes was the ChromHMM (Core 15-state model); the mammalian conservation algorithms GERP and SiPhy-omega, and positions are shown relative to GENCODE genes (Supplementary Data 1). HaploReg v4.1 includes GWAS and eQTL (including GTEx) and GRASP eQTL updates which were queried for the SNPs and included in Supplementary Data 1.

We also queried the GTEx portal and ExSNP integrated eQTL databases^21^ for the top candidate genes (based on loci identified in this study and similar function in early pregnancy reported previously^30,32^, *GDF15* and *IGFBP7*) to identify eQTLs (17 and 28 studies, respectively) included in the databases. The Genotype-Tissue Expression (GTEx) Project was supported by the Common Fund of the Office of the Director of the National Institutes of Health, and by NCI, NHGRI, NHLBI, NIDA, NIMH, and NINDS. The data used for the analyses described in this manuscript were obtained from (https://gtexportal.org/home/bubbleHeatmapPage/) the GTEx Portal on 05 May 2017. The SNPs identified were cross checked with the SNPs identified in haplogroups by HaploReg.

Functional annotation of all SNPs (SNPs *r*^2^ > 0.2 with SNPs in the two loci that were found to be in coding regions by HaploReg were queried for all predicted consequences using the 1000 Genomes Project^33^.

Finally, published studies reporting SNPs linked to altered expression of the top candidate genes (*GDF15* and *IGFBP7)* and SNPs in the top candidate genes reported in association studies in the GWAS catalog (http://www.ebi.ac.uk/gwas/ accessed on May 2017) and/or in published studies were cross checked with the SNPs identified in HaploReg to determine whether they are in LD with the SNPs linked to HG in our study.

### Code availability

All relevant software, including version details, used for the primary GWAS and conditional analyses are described in the references included in the Methods section. All relevant codes with respect to the replication are available from the author M.S.F. (mfejzo@mednet.ucla.edu).

### Data availability

Qualified researchers can contact apply.research@23andMe.com to gain access to full GWAS summary statistics following an agreement with 23andMe that protects 23andMe participant privacy. All relevant replication data are available from the author M.S.F. (mfejzo@mednet.ucla.edu).

## Electronic supplementary material

Supplementary Information(PDF 4391 kb)

Description of Additional Supplementary Files(PDF 799 kb)

Supplementary Data 1(XLSX 99 kb)

Supplementary Data 2(XLSX 99 kb)
