# Supplementary material for: Placenta and appetite genes GDF15 and IGFBP7 are associated with hyperemesis gravidarum
Source: Nat Commun. 2018 Mar 21;9:1178. doi: 10.1038/s41467-018-03258-0 (PMC5862842; doi:10.1038/s41467-018-03258-0)
Supplement: Supplementary file 1 — Supplementary Information(PDF 4391 kb) [file 41467_2018_3258_MOESM1_ESM.pdf]

Supplementary information for

Placenta and appetite genes *GDF15* and *IGFBP7* are associated with hyperemesis gravidarum

Fejzo et al.

Supplementary Figure 1. Quantile-quantile plots for a) SCAN1 (binary phenotype) and b) SCAN2 (ordinal phenotype).

a)

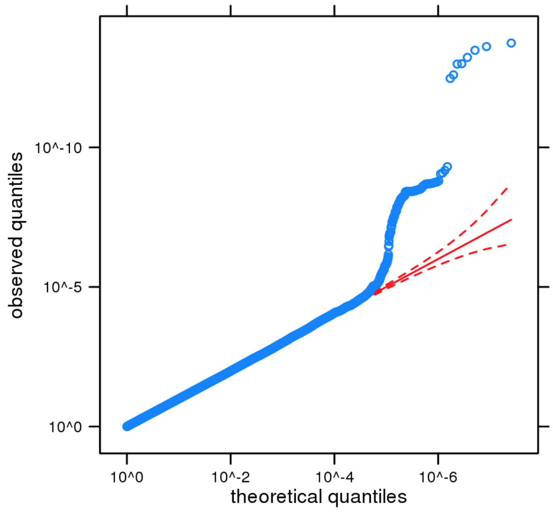

b)

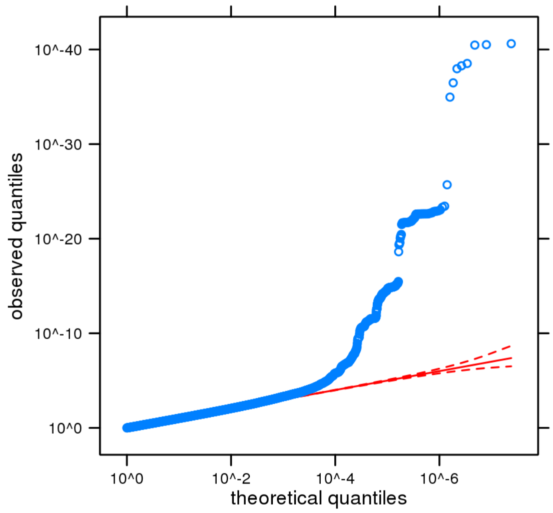

Supplementary Figure 2. LocusZoom plots for loci with *P*-values < 10^{-6} in SCAN1.

a) chr19p13.11

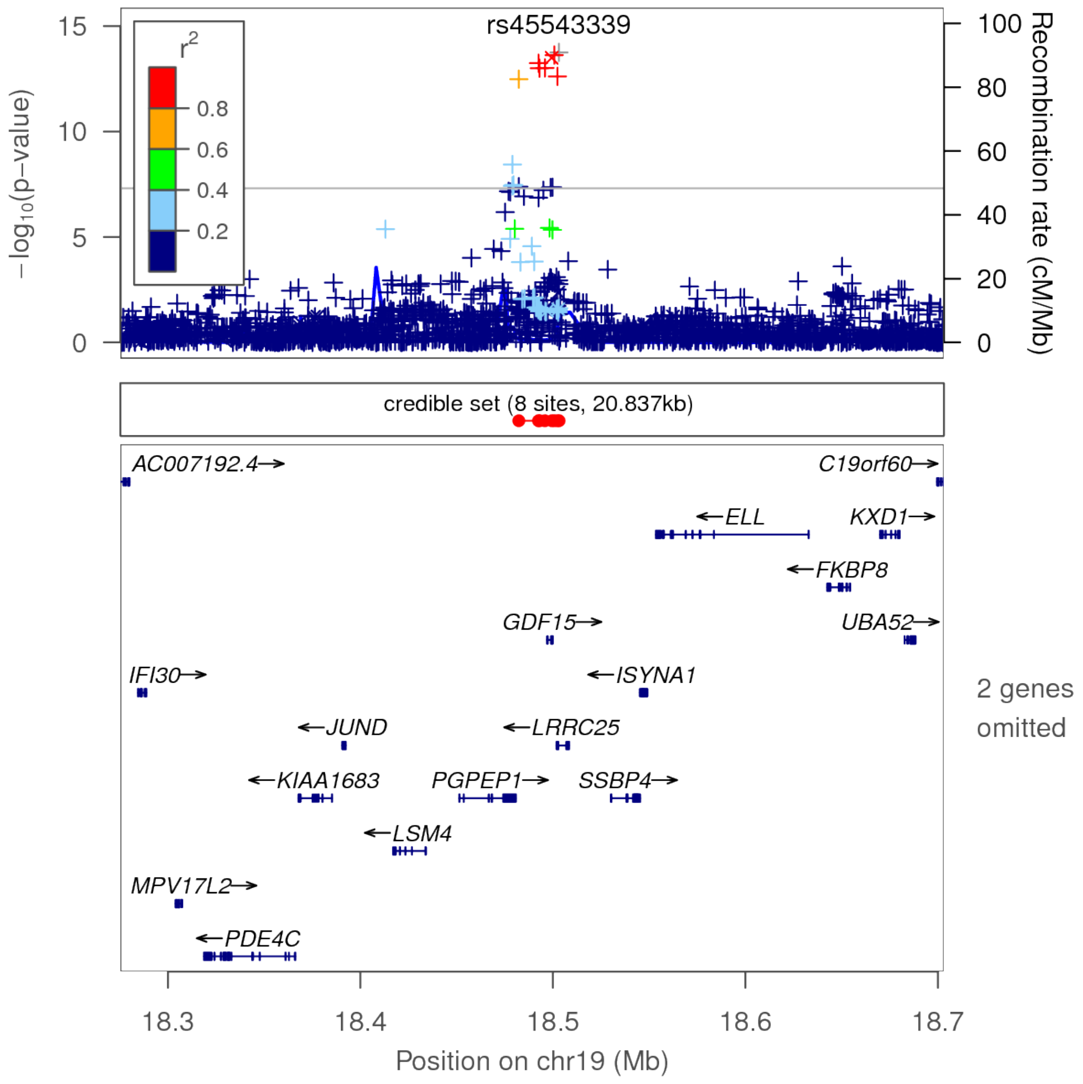

b) chr4q12

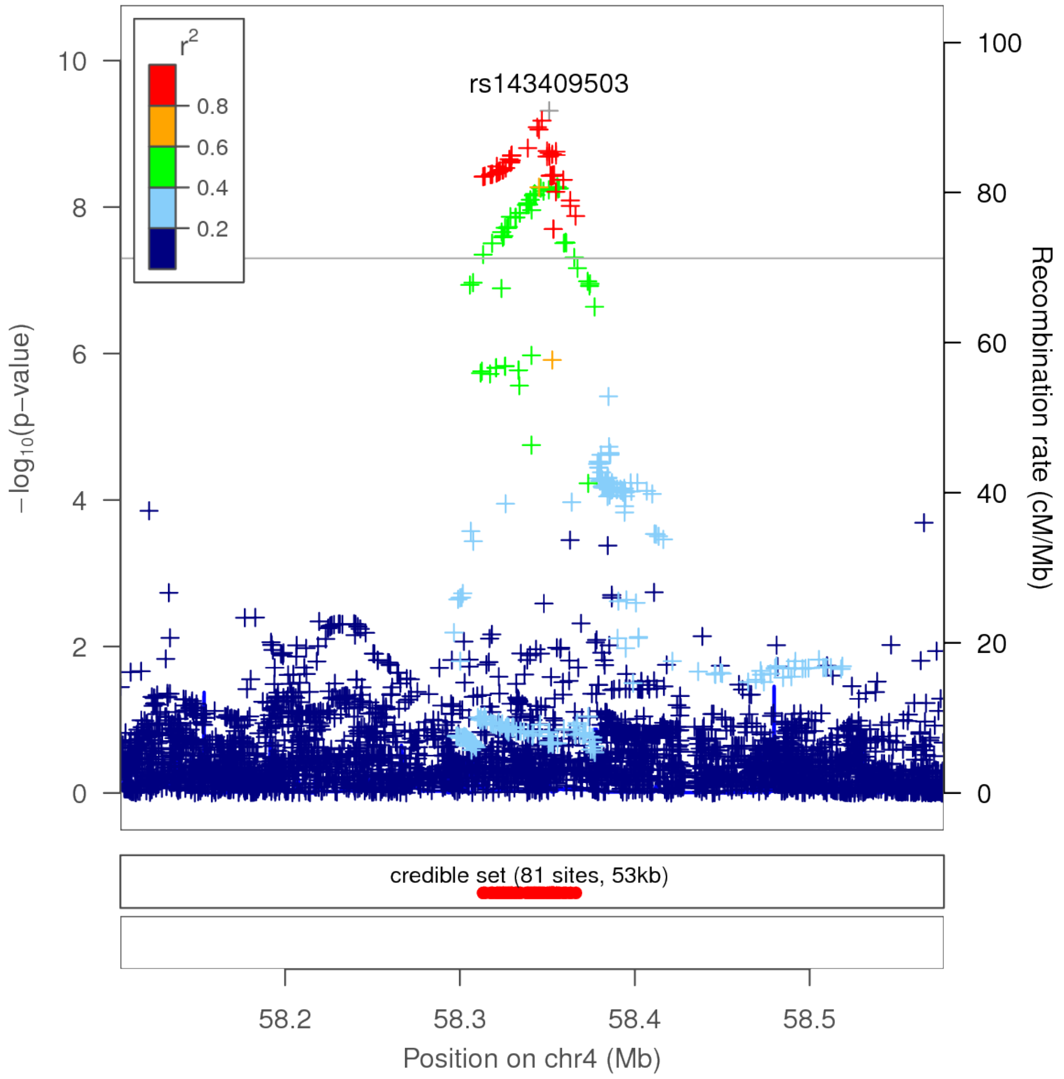

r2
0.8
0.6
0.4
0.2
rs143409503
10
8
6
4
2
0
−log10(p−value)
100
80
60
40
20
0
Recombination rate (cM/Mb)
credible set (81 sites, 53kb)
58.2
58.3
58.4
58.5
Position on chr4 (Mb)

c) 22q12.3

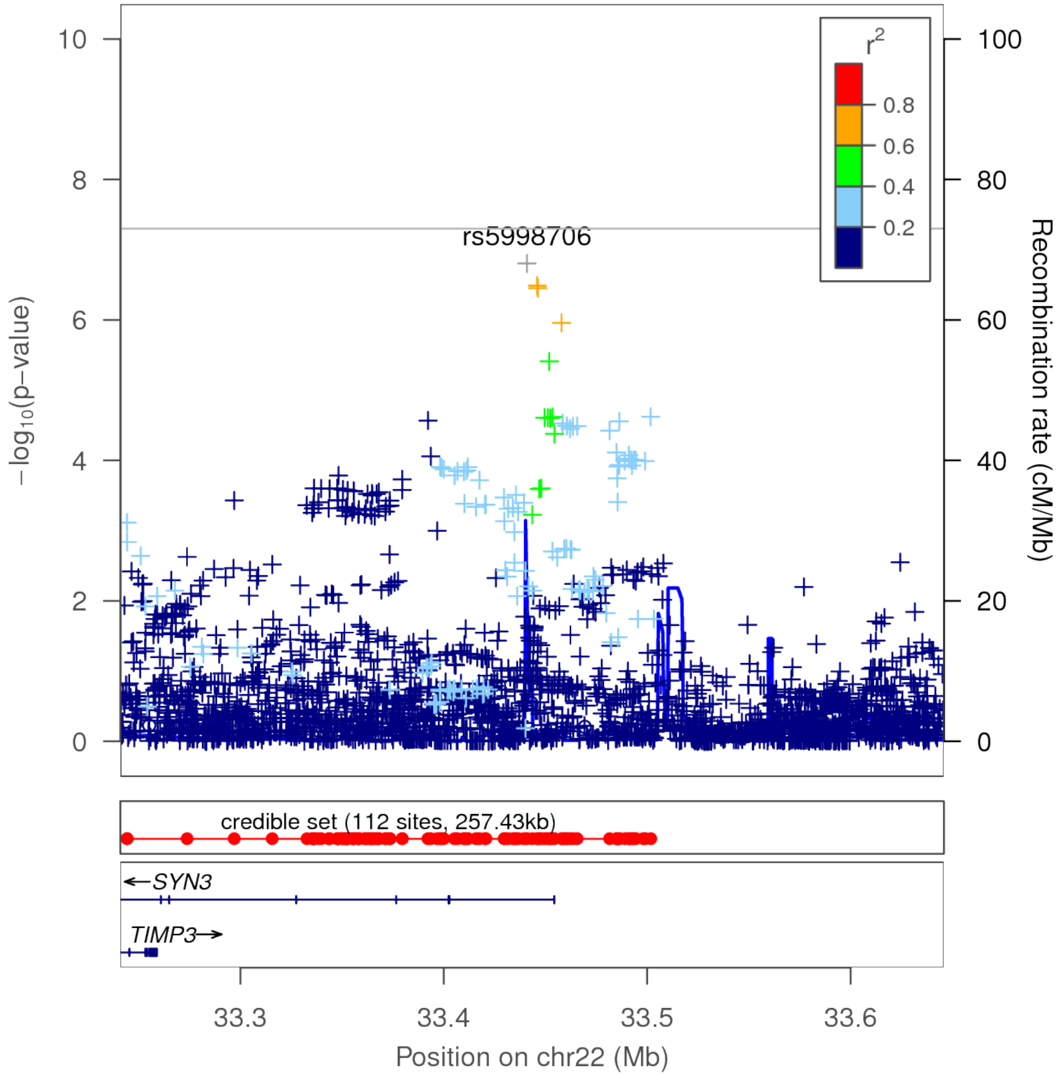

rs5998706
r2
0.8
0.6
0.4
0.2
−log10(p−value)
Recombination rate (cM/Mb)
credible set (112 sites, 257.43kb)
SYN3
TIMP3
33.3
33.4
33.5
33.6
Position on chr22 (Mb)

d) chr2q33.3

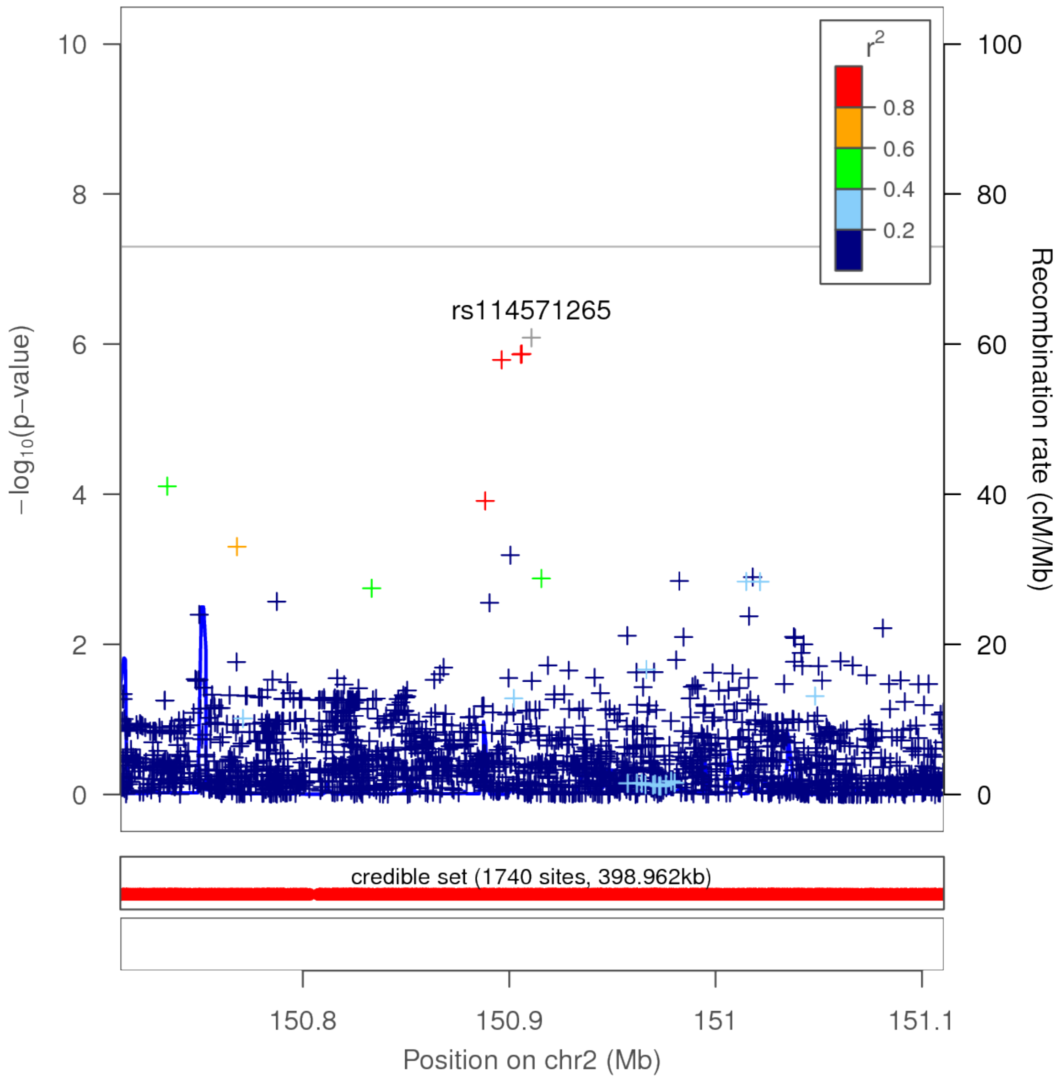

10
8
6
4
2
0
−log10(p−value)
100
80
60
40
20
0
Recombination rate (cM/Mb)
r2
0.8
0.6
0.4
0.2
rs114571265
credible set (1740 sites, 398.962kb)
150.8
150.9
151
151.1
Position on chr2 (Mb)

e) chr9q31.2

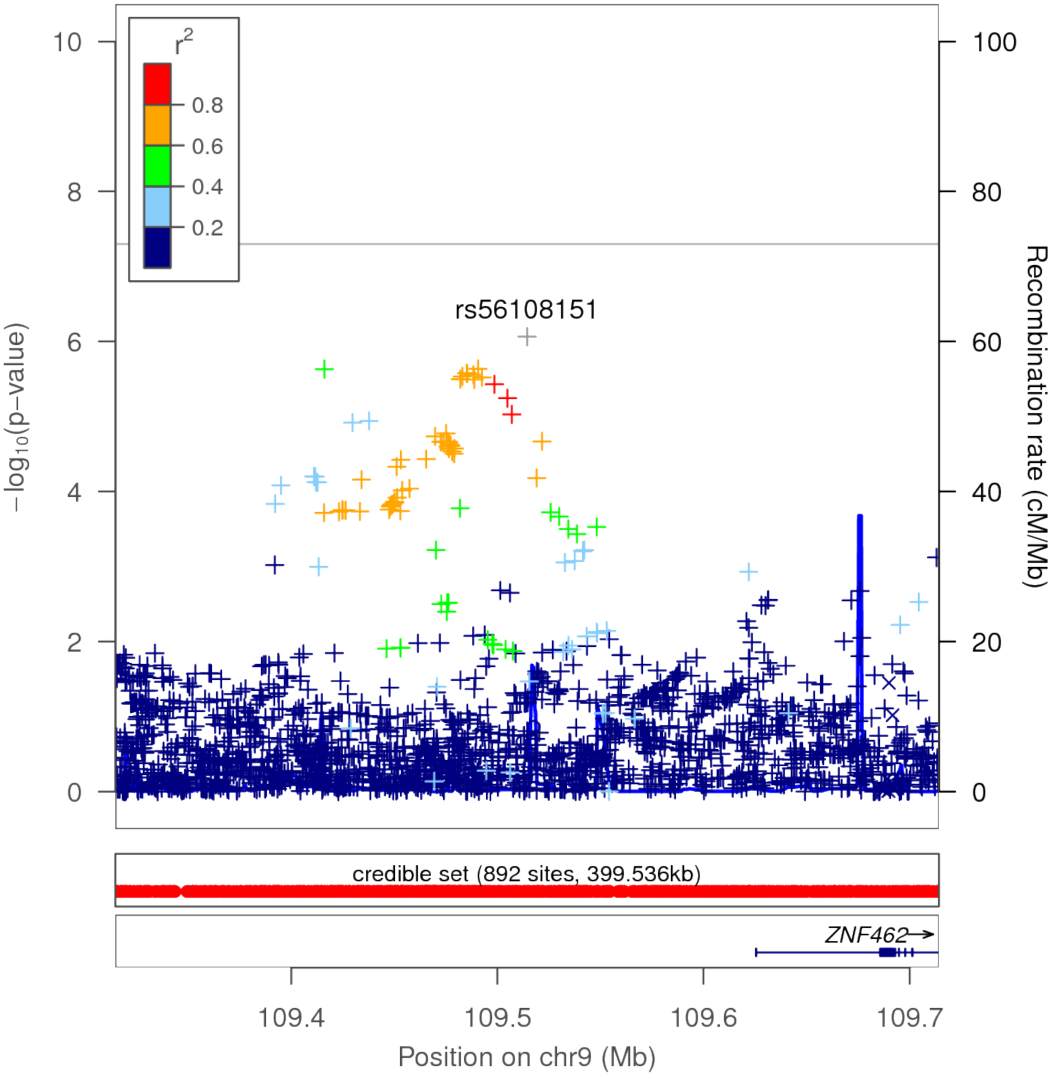

r2
0.8
0.6
0.4
0.2
10
8
6
4
2
0
100
80
60
40
20
0
−log10(p−value)
Recombination rate (cM/Mb)
rs56108151
credible set (892 sites, 399.536kb)
ZNF462
109.4
109.5
109.6
109.7
Position on chr9 (Mb)

Supplementary Figure 3. LocusZoom plots for loci with *P*-values < $5x10^{-8}$ in SCAN2.

a) chr19p13.11

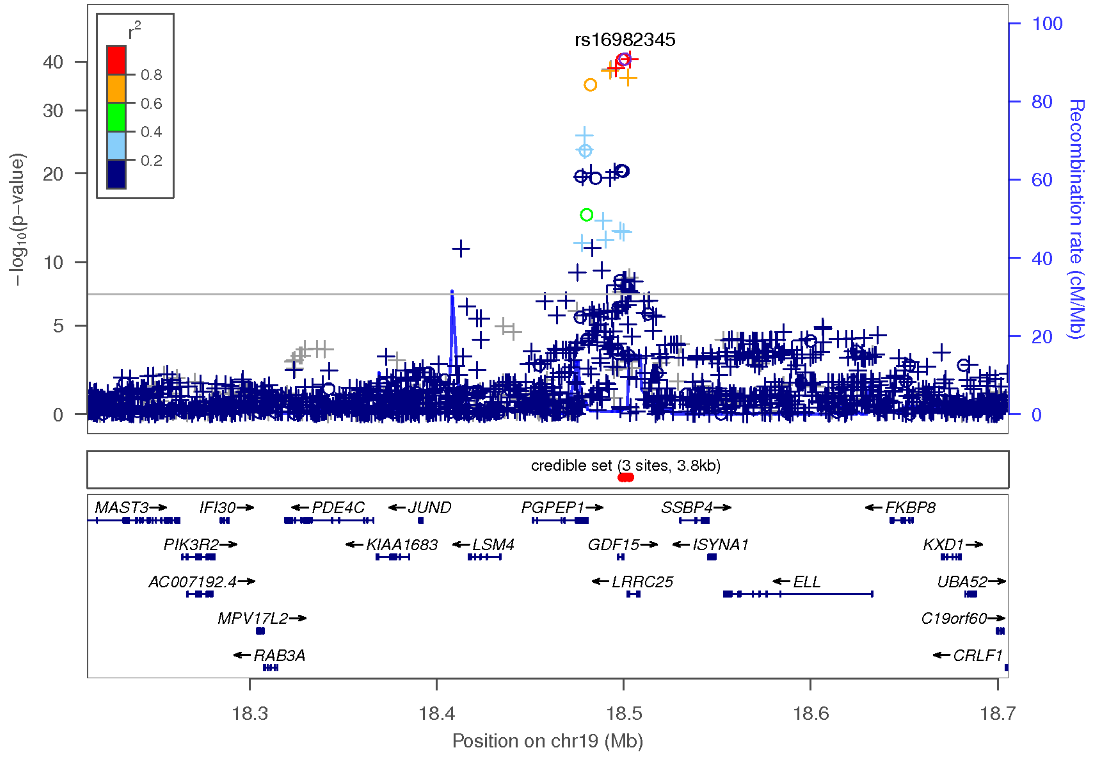

b) chr*4q12*

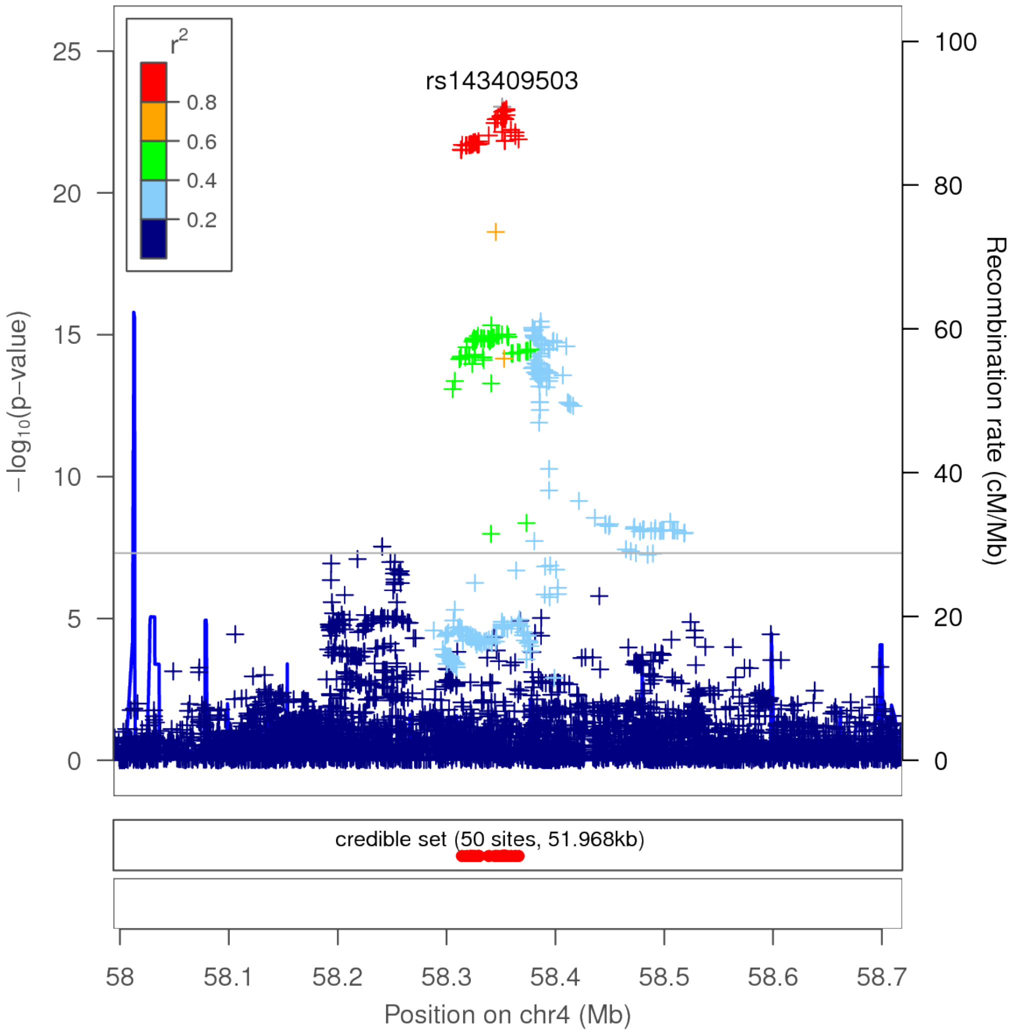

r2
0.8
0.6
0.4
0.2
rs143409503
25
20
15
10
5
0
−log10(p−value)
100
80
60
40
20
0
Recombination rate (cM/Mb)
credible set (50 sites, 51.968kb)
58
58.1
58.2
58.3
58.4
58.5
58.6
58.7
Position on chr4 (Mb)

c) 11q22.1

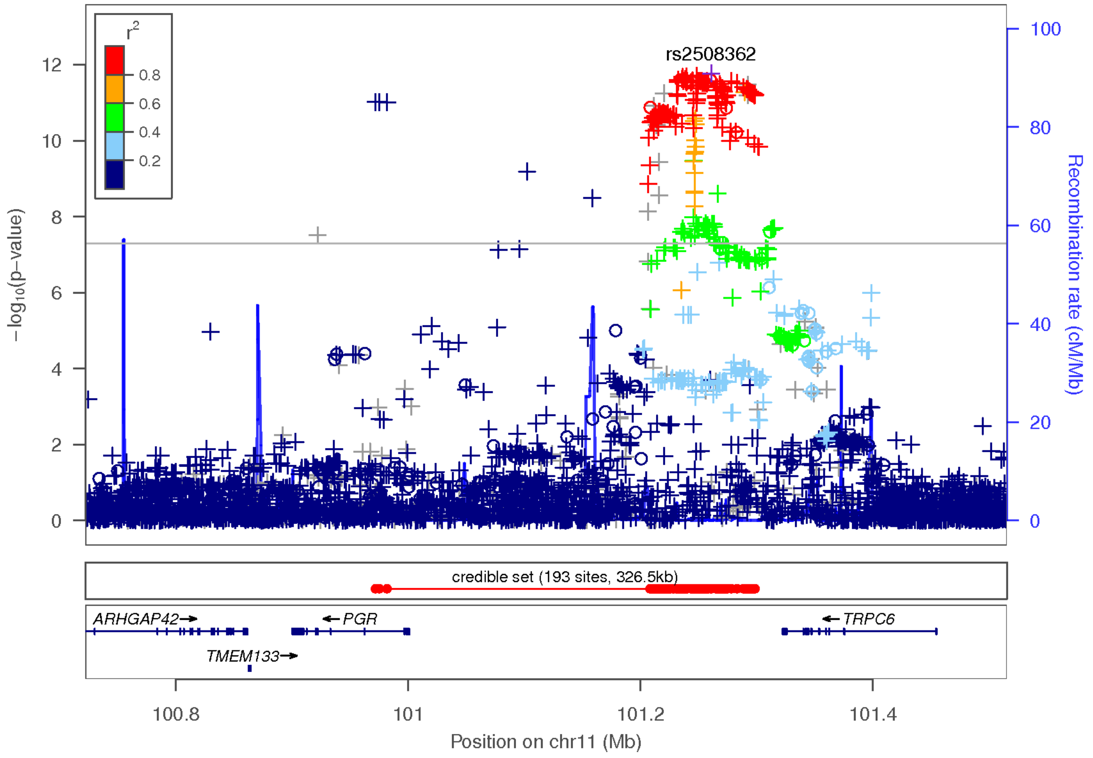

r²
0.8
0.6
0.4
0.2
rs2508362
−log10(p−value)
Recombination rate (cM/Mb)
credible set (193 sites, 326.5kb)
ARHGAP42
PGR
TMEM133
TRPC6
100.8
101
101.2
101.4
Position on chr11 (Mb)

d) chr6p15

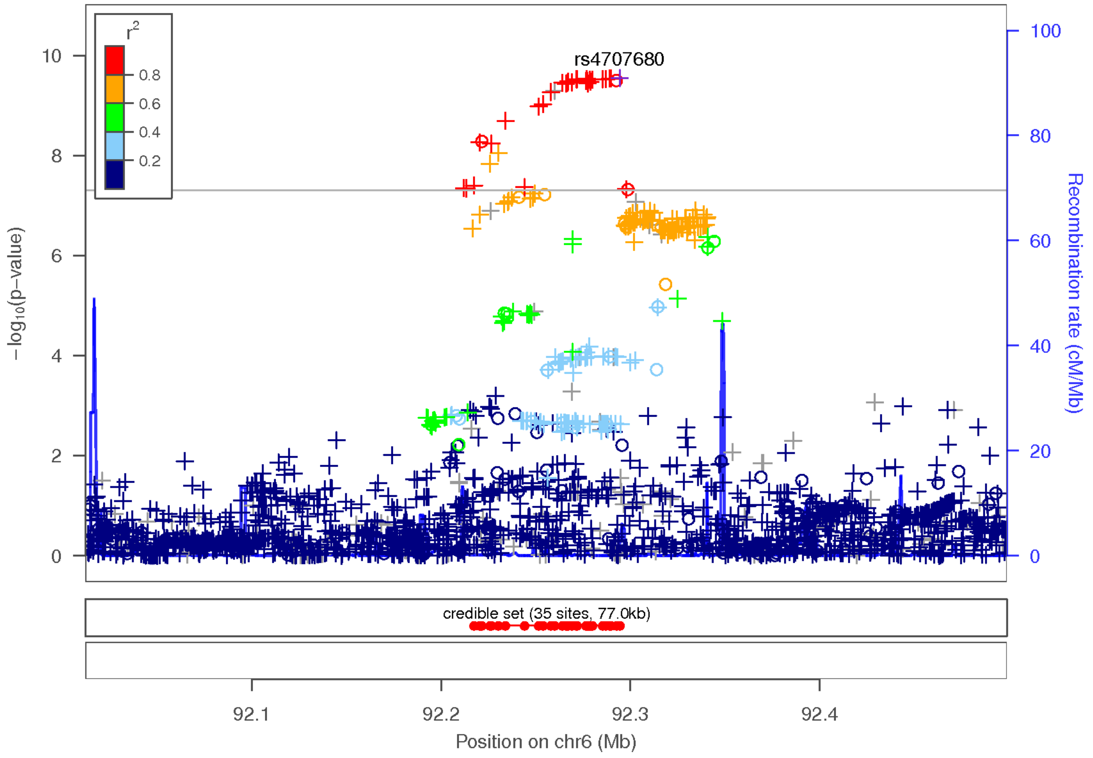

r²
0.8
0.6
0.4
0.2
rs4707680
10
8
6
4
2
0
−log₁₀(p−value)
100
80
60
40
20
0
Recombination rate (cM/Mb)
credible set (35 sites, 77.0kb)
92.1
92.2
92.3
92.4
Position on chr6 (Mb)

e) 22q11.23

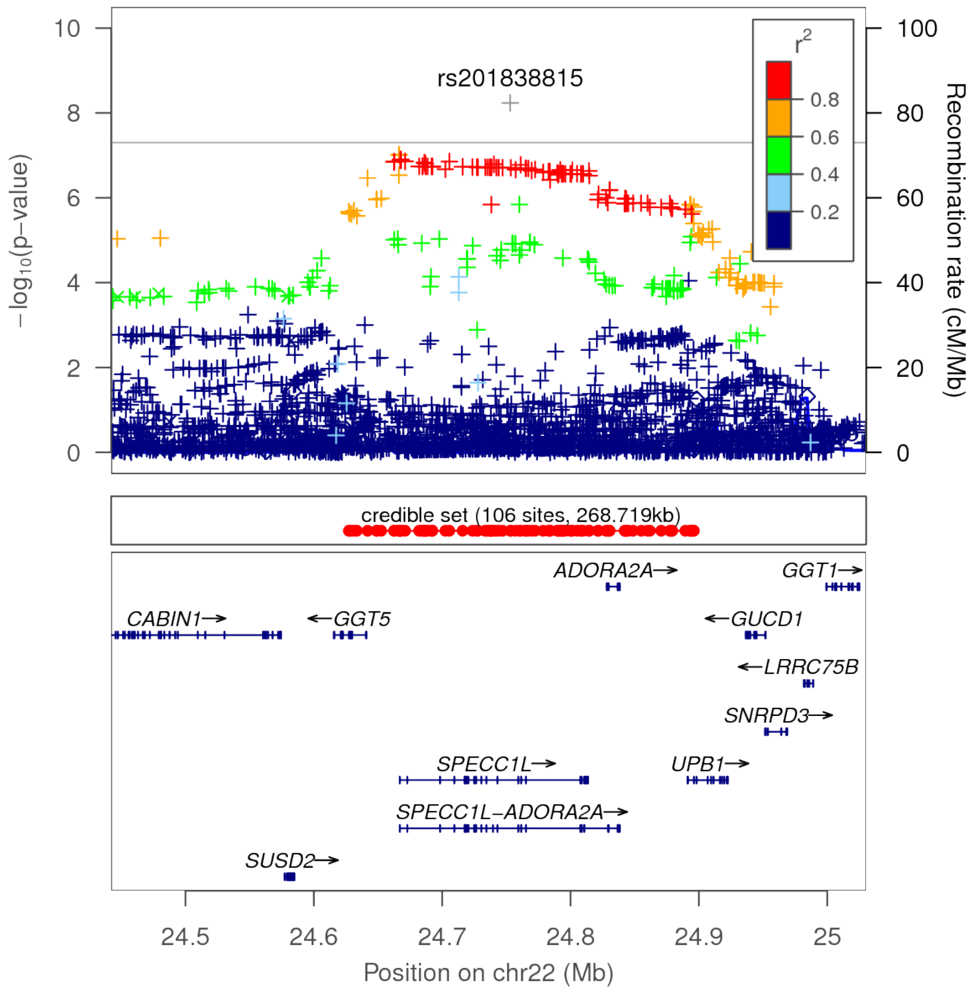

rs201838815
r2
0.8
0.6
0.4
0.2
−log10(p−value)
Recombination rate (cM/Mb)
credible set (106 sites, 268.719kb)
ADORA2A→
GGT1→
CABIN1→
←GGT5
←GUCD1
←LRRC75B
SNRPD3→
SPECC1L→
UPB1→
SPECC1L−ADORA2A→
SUSD2→
24.5
24.6
24.7
24.8
24.9
25
Position on chr22 (Mb)

f) 6p12.1

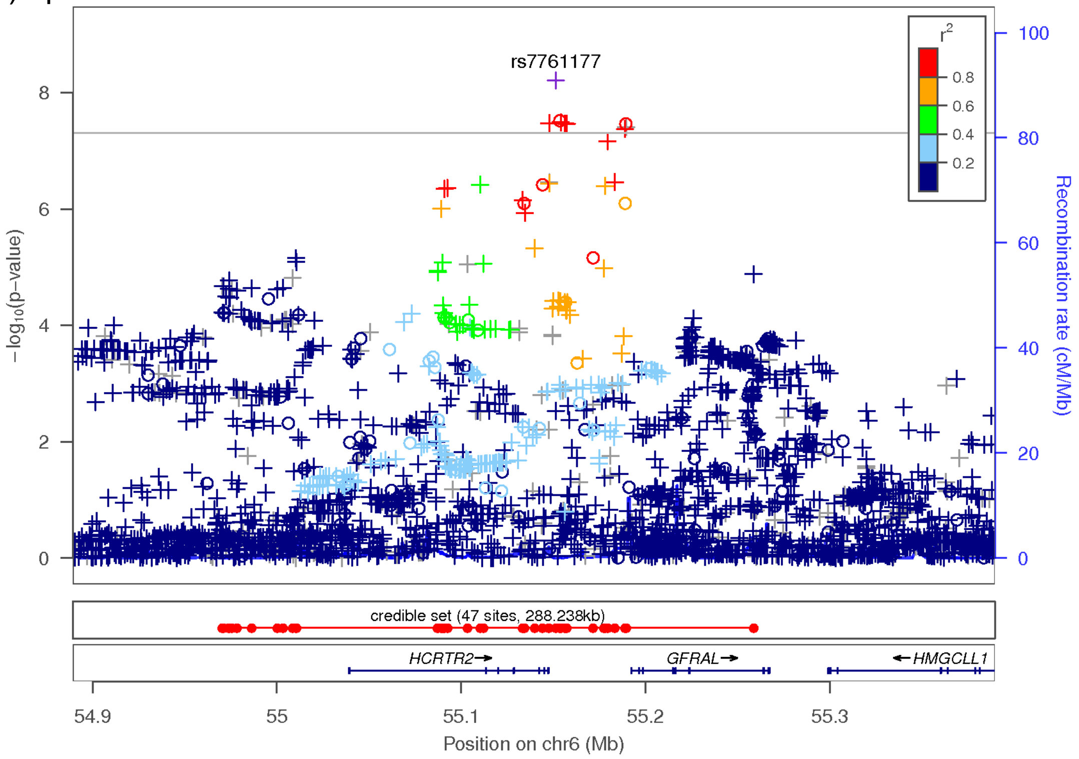
rs7761177
r2
0.8
0.6
0.4
0.2
−log10(p−value)
Recombination rate (cM/Mb)
credible set (47 sites, 288.238kb)
HCRTR2
GFRAL
HMGCLL1
54.9
55
55.1
55.2
55.3
Position on chr6 (Mb)

g) chr4q31.12

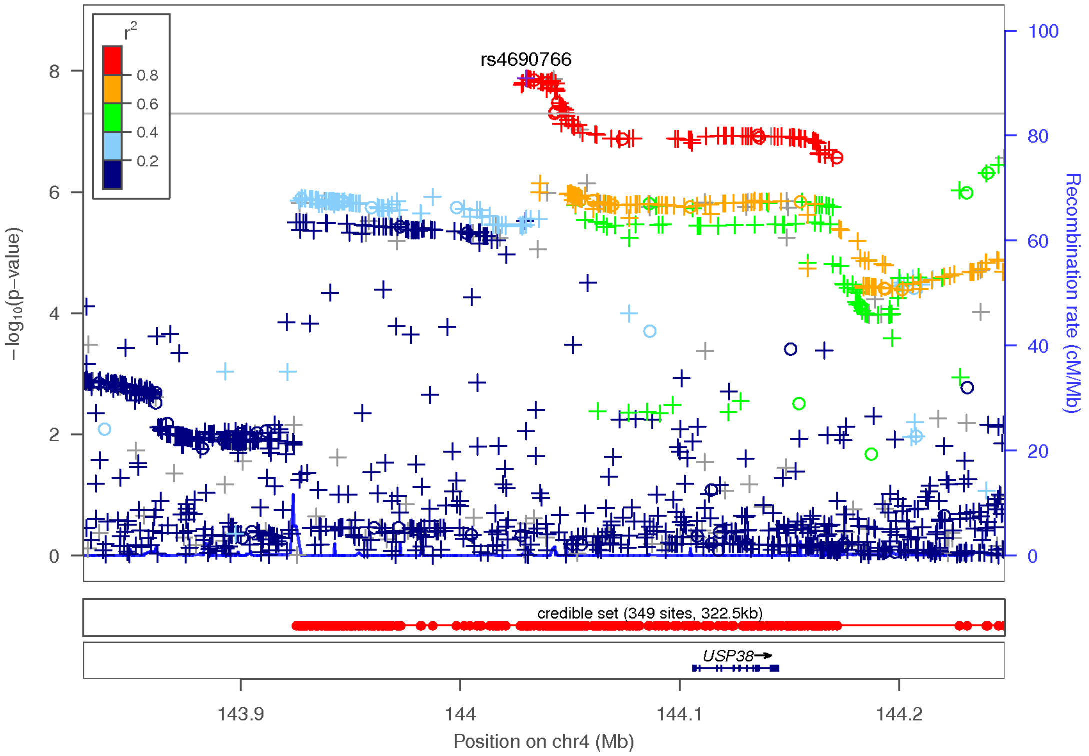

r2
0.8
0.6
0.4
0.2
rs4690766
8
6
4
2
0
−log10(p−value)
100
80
60
40
20
0
Recombination rate (cM/Mb)
credible set (349 sites, 322.5kb)
USP38
143.9
144
144.1
144.2
Position on chr4 (Mb)

Supplementary Figure 4. LocusZoom plots illustrating stepwise conditional regression in (a) SCAN1
and (b) SCAN2, at the chr19p13.11 locus.

(a) SCAN1

Step 1: The GWAS signal, best SNP is rs45543339, p = 1.8×10^{-14}

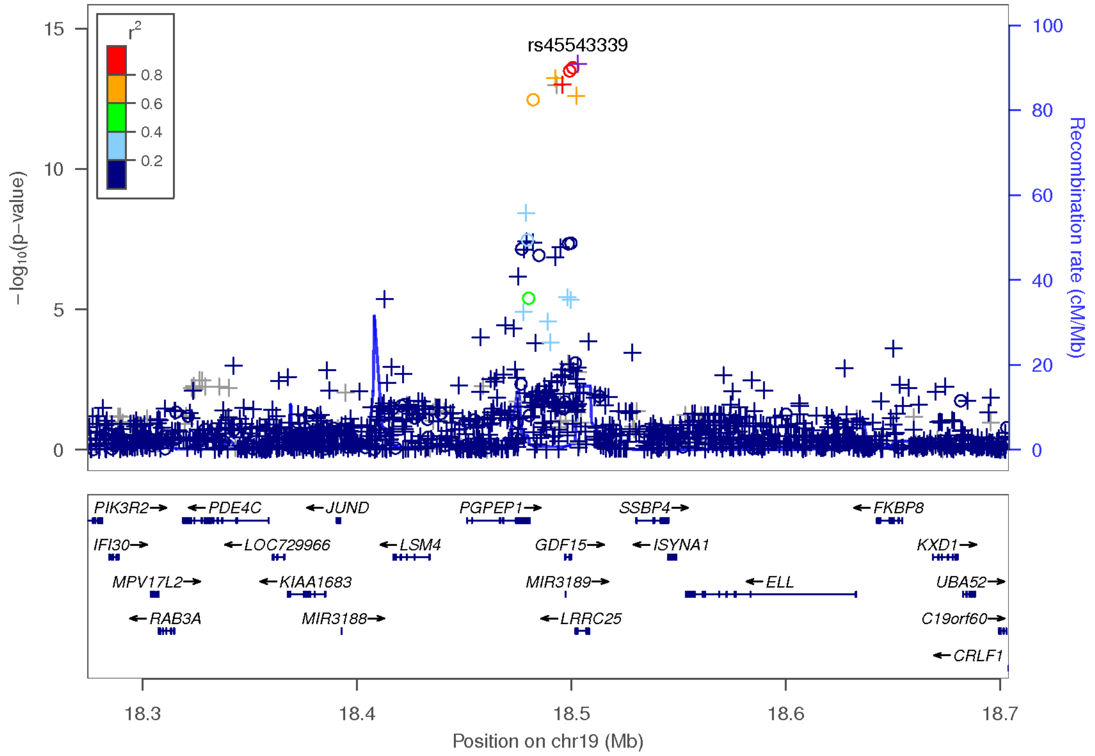

Step 2: The GWAS signal, best SNP is rs1054221, $p = 1.7x10^{-7}$

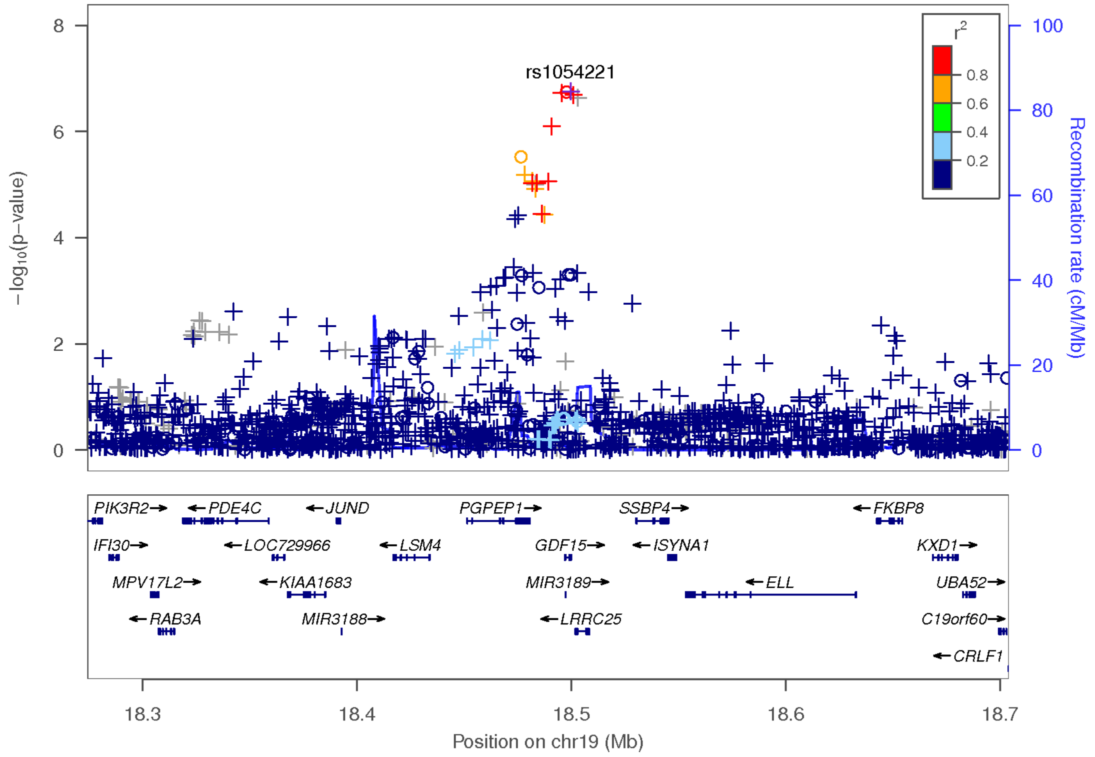

Step 3: No significant association signals at $p<10^{-5}$

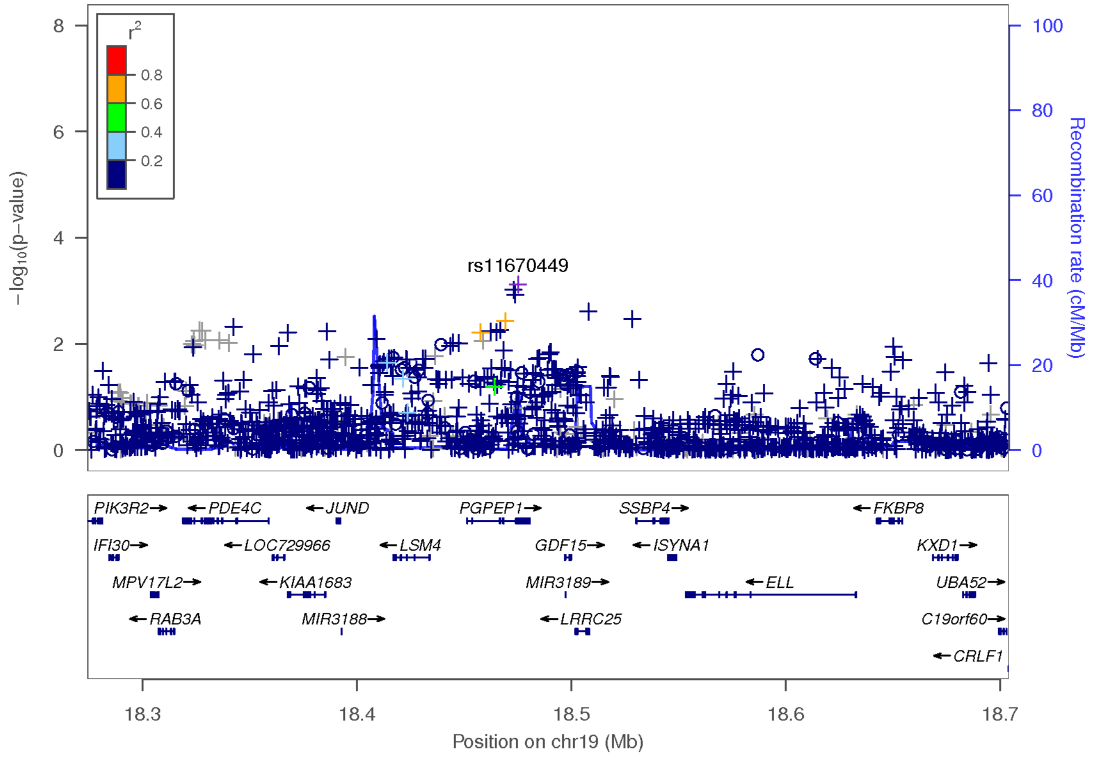

(b) SCAN2

Step 1: The GWAS signal, best SNP is rs16982345, p = 2.4×10^{-41}

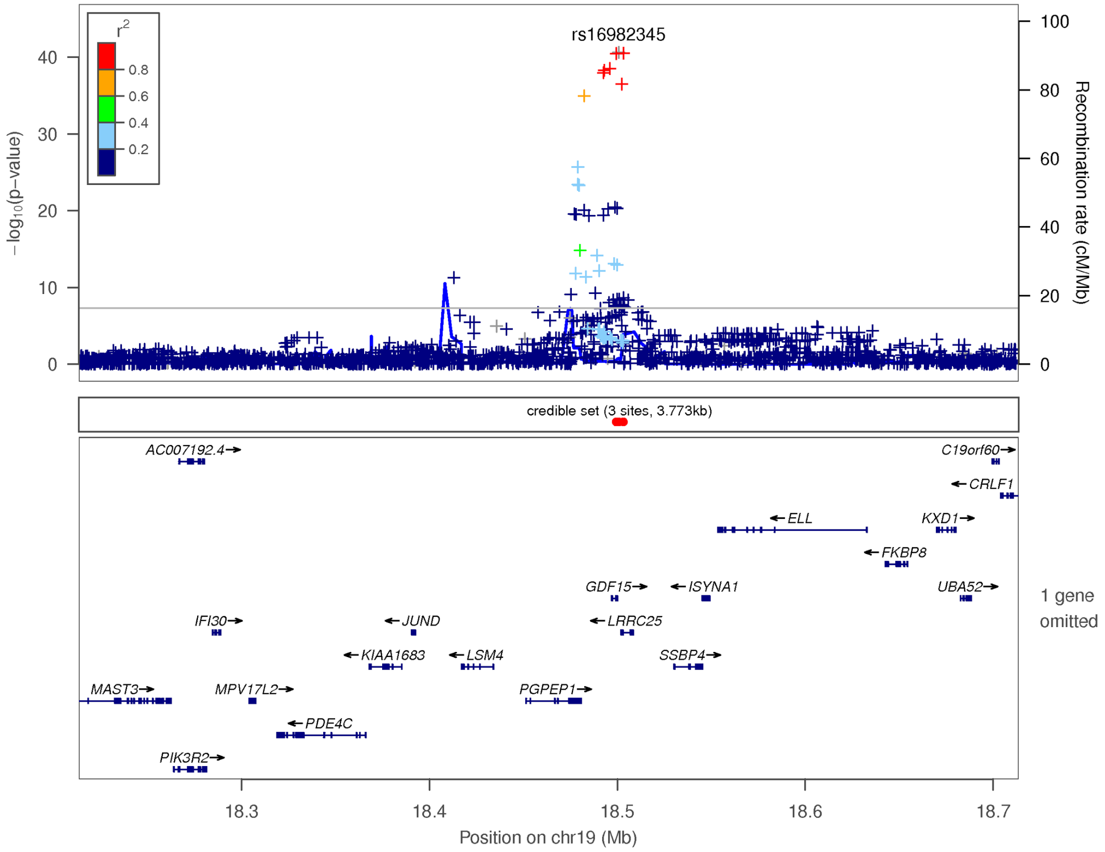

Step 2: Best SNP is rs34345957, p = $3.5x10^{-21}$

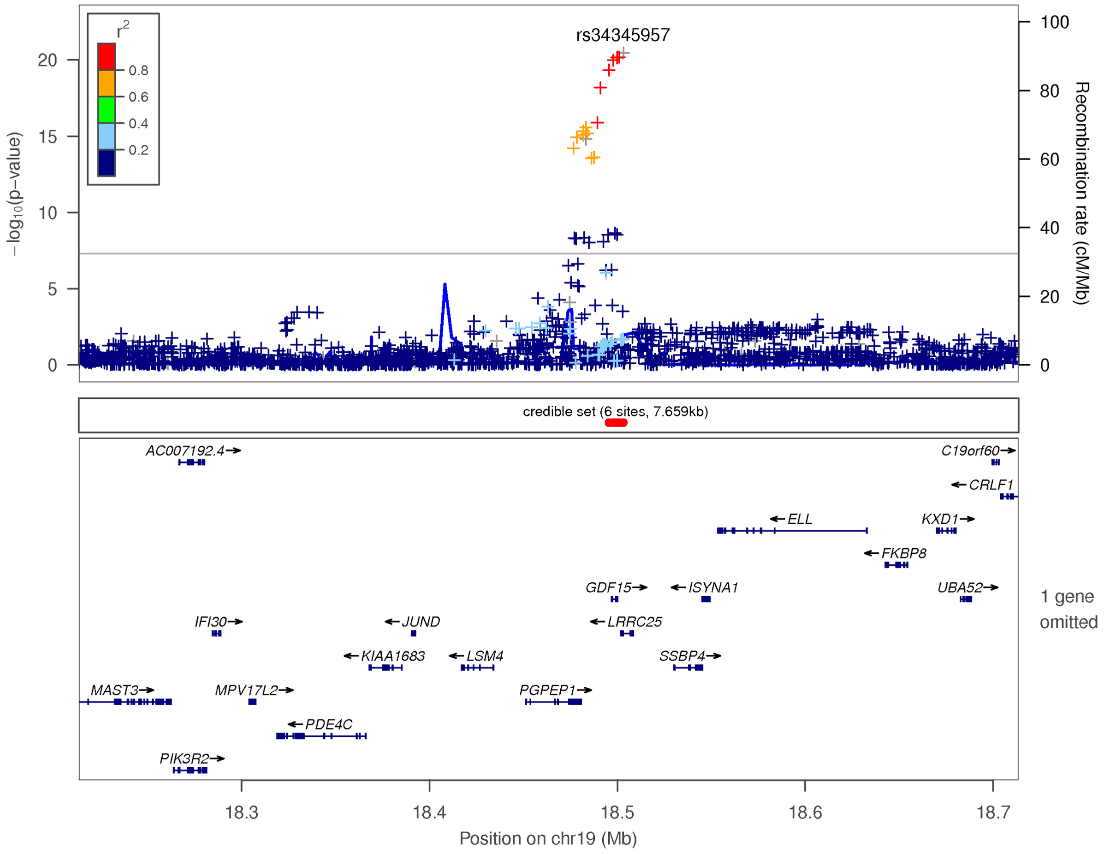

Step 3: Best SNP is rs4808787, $p = 2.1 \times 10^{-6}$

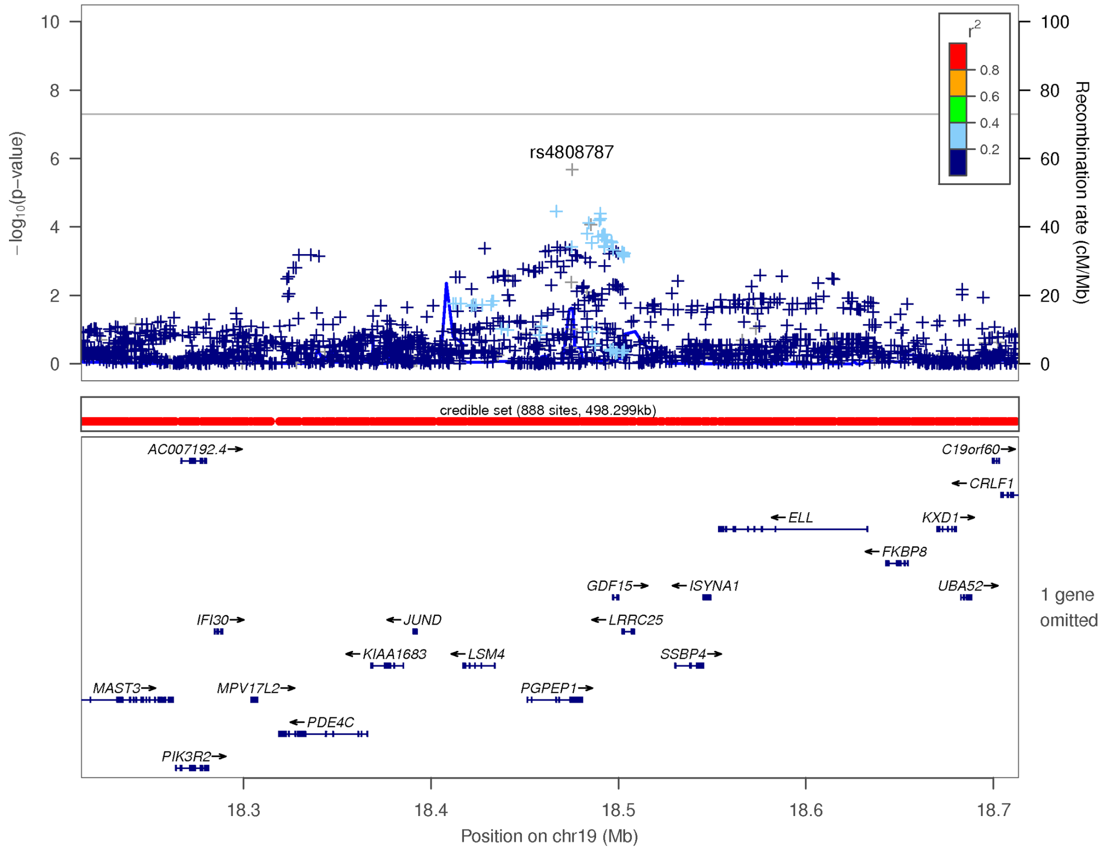

Step 4: No significant association signals at $p<10^{-5}$

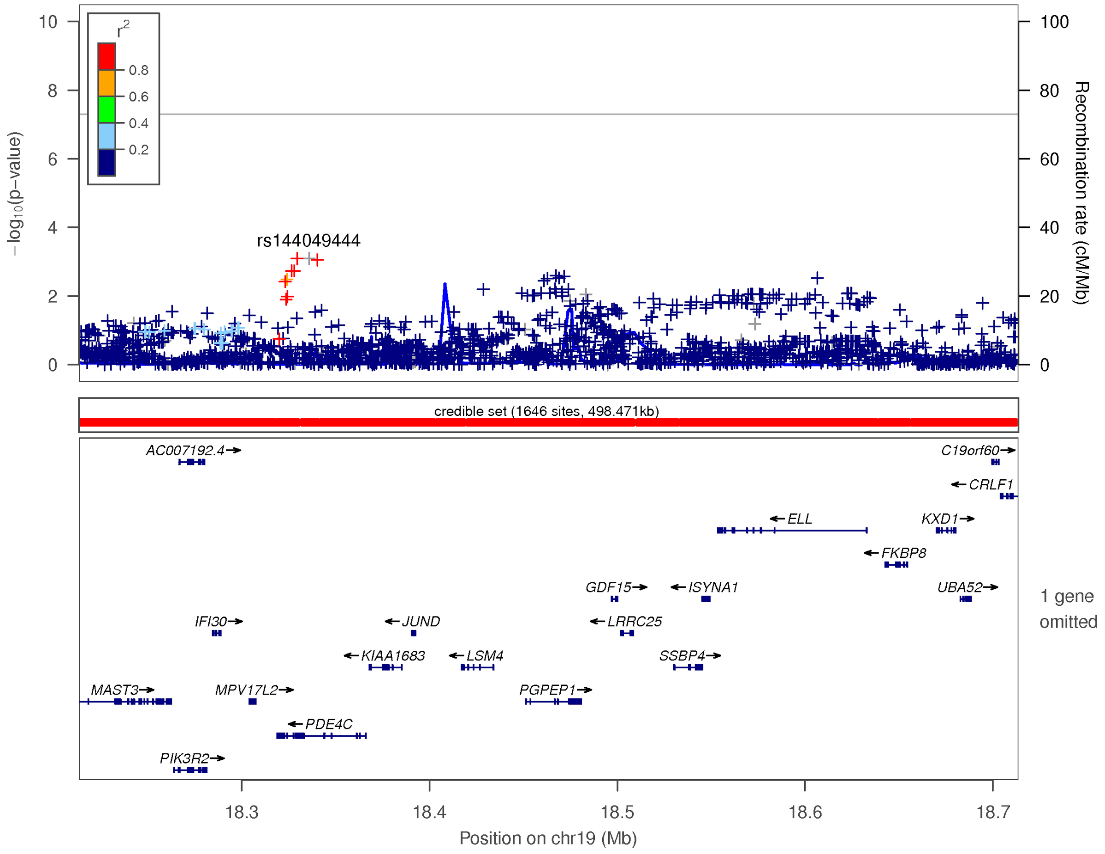

Supplementary Figure 5. LocusZoom plots illustrating stepwise conditional regression in SCAN2, at the 11q22.1 locus.

Step 1: The GWAS signal, best SNP is rs2508362, $p = 1.7 \times 10^{-12}$

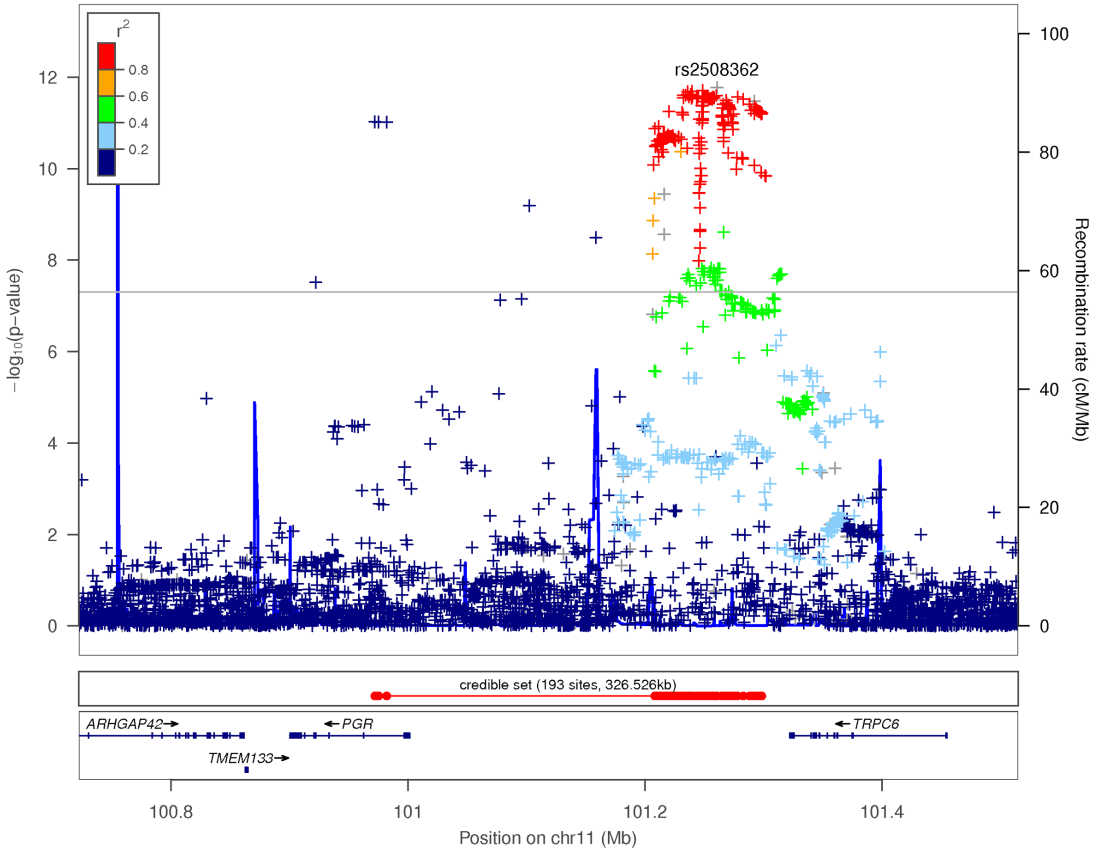

Step 2: Best SNP is rs78025546, p = $1.1x10^{-12}$

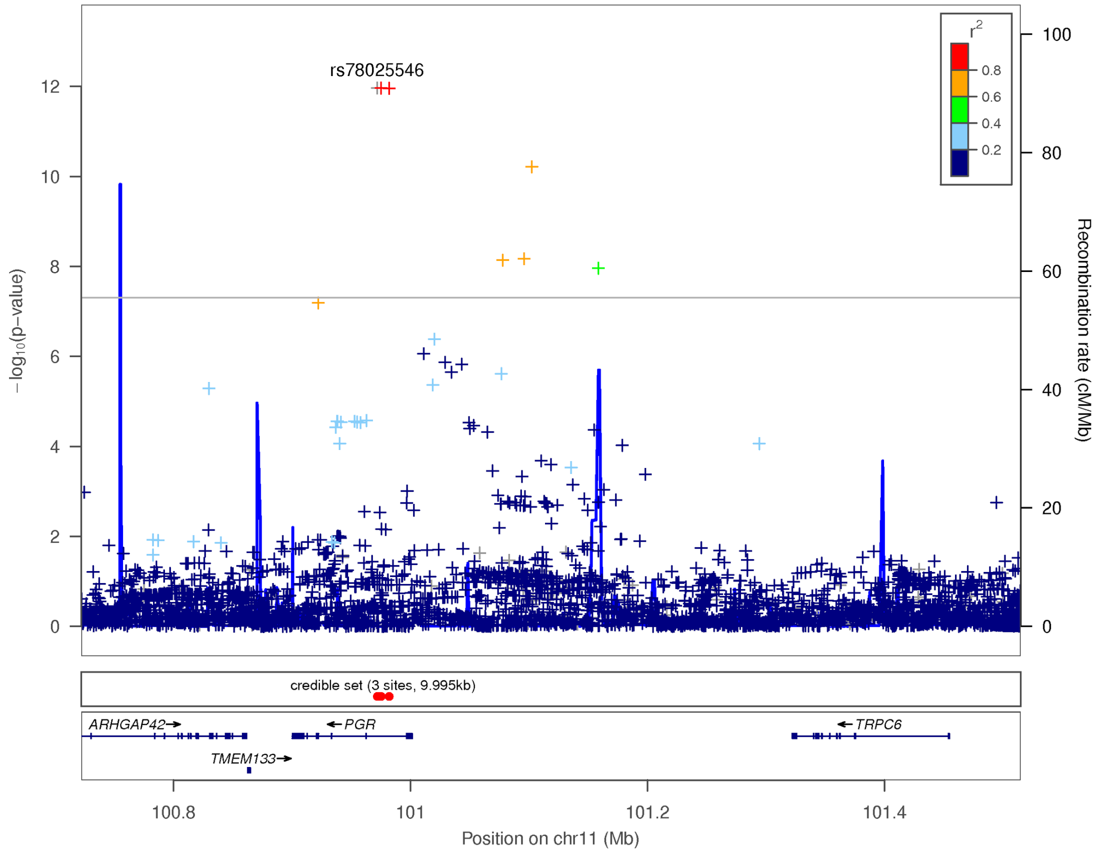

Step 3: No significant association signals at $p<10^{-5}$

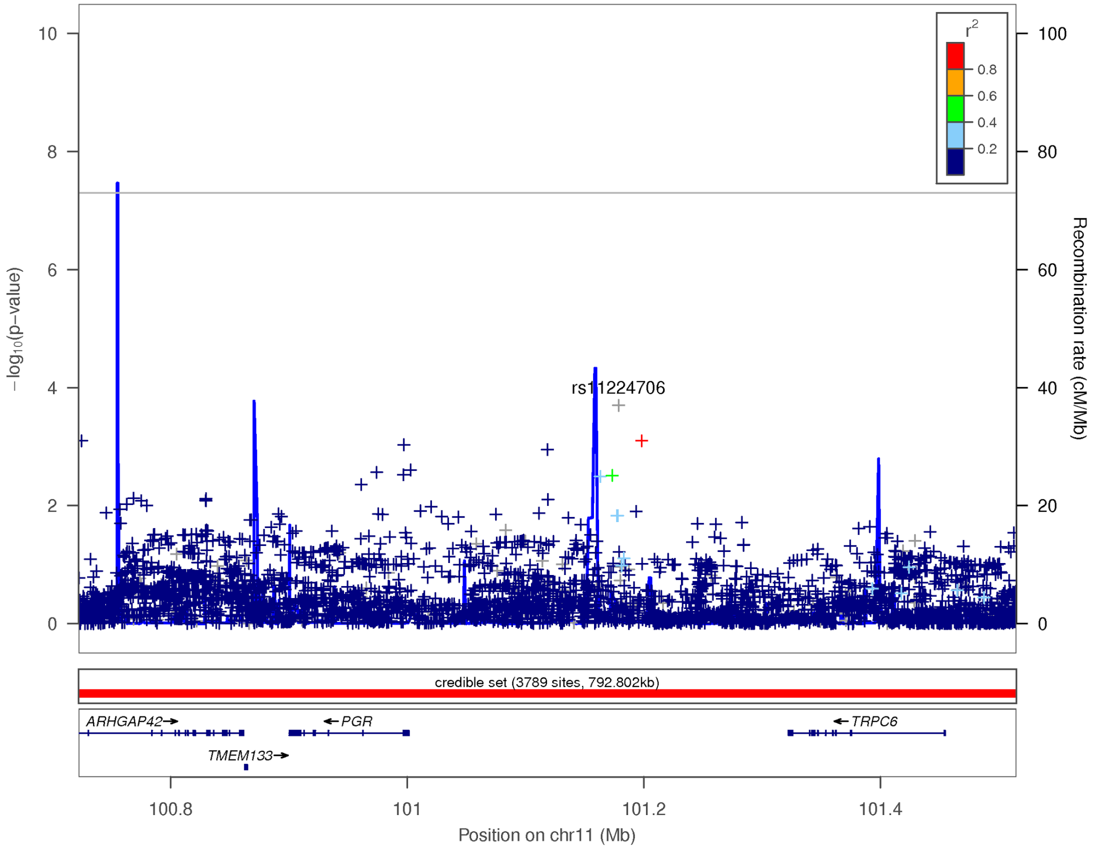

Supplementary Table 1. Cohort overlap statistics (a) and SNP-level QC information for b) SCAN1 and c) SCAN2.

a) Individuals included in both SCAN1 and SCAN2 cohorts. Exact counts of 5 or fewer individuals were masked to protect the privacy of 23andMe customers.

**Phenotype**	**SCAN1 - Case**	**SCAN1 - Control**
**SCAN2 - “None”**	0	14,944
**SCAN2 - “Slight”**	0	0
**SCAN2 - “Moderate”**	≤ 5	0
**SCAN2 - “Severe”**	≤ 5	0
**SCAN2 - “Very Severe”**	1,216	0

b)

Results for QC filters on genotyped data

	**failed**	**passed**
no filters	0	1050074
not V/V2-only, chrM, chrY	34319	1015755
parent-offspring test	6291	1009544
MAF> 0%	3510	1006267
HWE > 1e-20	59234	948340
gt.rate > 90%	29643	931193
batch effects	39525	921199

Results for QC filters on imputed dosage data

	**failed**	**passed**
no filters	0	15574220
MAF> 0%	0	15574220
imputation quality	2502882	13071338
batch effects	65	13071338

Results for QC filters on merged association test results

	**failed**	**passed**
imputed only	12224646	12224646
both passed	846234	13070880

genotyped only	74965	13145845
no test result	−35599	13110246
failed to converge	−422362	12687884

c)

Results for QC filters on genotyped data

	failed	passed
no filters	0	1050074
not V/V2-only, chrM, chrY	34319	1015755
parent-offspring test	6291	1009544
MAF> 0%	3510	1006267
HWE > 1e-20	59234	948340
gt.rate > 90%	29643	931193
batch effects	39525	921199

Results for QC filters on imputed dosage data

	failed	passed
no filters	0	15574220
MAF> 0%	0	15574220
imputation quality	2502882	13071338
batch effects	65	13071328

Results for QC filters on merged association test results

	failed	passed
imputed only	12224646	12224646
both passed	846234	13070880
genotyped only	74965	13145845
no test result	−23768	13122077
failed to converge	−1165146	11956931

Supplementary Table 2. Association signals in SCAN2 with $5x10^{-8}$ < p-value < 10^{-6}.

marker	position	alleles	MAF	*P*-value	β	95% CI	Gene context
rs2076308	6:50791640	G/C	0.184	2.7×10^{-7}	0.045	[0.028, 0.062]	[TFAP2B]
rs11793821	9:86184504	A/G	0.14	3.0×10^{-7}	−0.050	[-0.069,-0.031]	FRMD3--[ ]--IDNK
rs35970726	22:33481511	A/-	0.319	3.2×10^{-7}	0.037	[0.023, 0.052]	SYN3--[ ]---LARGE
rs17077506	13:84508874	C/G	0.134	6.9×10^{-7}	−0.049	[-0.068,-0.030]	SLITRK1--[ ]
rs148644662	4:157658837	G/T	0.001	7.0×10^{-7}	0.636	[0.385,0.888]	CTSO---[ ]--PDGFC
rs11398016	21:22910644	-/T	0.475	7.3×10^{-7}	−0.033	[-0.046,-0.020]	[NCAM2]
rs12992546	2:237918501	T/C	0.341	7.5×10^{-7}	0.035	[0.021, 0.049]	CXCR7---[ ]--COPS8
rs75481205	6:100228145	A/G	0.048	9.6×10^{-7}	−0.087	[-0.122,-0.052]	PRDM13---[ ]---MCHR2

Supplementary Table 3. Joint model results of step-wise conditional analysis for a) SCAN1 and b) SCAN2. Values are reported with respect to the first allele.

a)

locus	step	marker	position	alleles	*P*-value	OR [95% CI]	Gene context
chr19p13.11	1	rs45543339	19:18503194	C/T	$3.3x10^{-18}$	0.63 [0.563,0.696]	*[LRRC25]*
chr19p13.11	2	rs1054221	19:18499858	C/T	$1.7x10^{-07}$	1.38 [1.22,1.56]	*[GDF15]*

b)

locus	step	marker	position	alleles	*P*-value	β	95% CI	Gene context
chr19p13.11	1	rs16982345	19:18500722	A/G	$1.4x10^{-64}$	0.133	[0.118,0.148]	*GDF15/ LRRC25*
chr19p13.11	2	rs34345957	19:18503168	-/G	$1.1x10^{-28}$	0.108	[0.089,0.127]	*LRRC25*
chr19p13.11	3	rs4808787	19:18475285	A/G	$6.0\text{-}x10^{-6}$	-0.037	[-0.053,-0.021]	*PGPEP1*
11q22.1	1	rs2508362	11:101260798	A/G	$5.0x10^{-16}$	0.064	[0.049,0.080]	*PGR/ TRPC6*
11q22.1	2	rs78025546	11:100972022	A/G	$6.2x10^{-14}$	0.167	[0.124,0.211]	*PGR*

Supplementary Note 1

Studies of other phenotypes showing association with *GDF15* and *IGFBP7*

Other SNPs in *GDF15* have been associated with body mass index,[45] HDL cholesterol levels,[19] prostate cancer,[42,44] cardiovascular disease,[41,43] systemic lupus erythematosus and periodontitis (GWAS catalog http://www.ebi.ac.uk/gwas/, accessed May 2017). The only SNP at the *IGFBP7* locus reported in a previous GWAS, rs11133504, in weak LD with rs14309503 and rs4865234 (Supplementary Data 1), was an NHGRI/GBI GWAS association signal in ulcerative colitis.[46] Other SNPs in *IGFBP7* have been associated with age-related macular degeneration,[47,48] AgG glycosylation,[49] opoioid dependence in males,[50] cotinine glucuronidation,[51] head and neck cancer risk,[52] bisphosophonate-induced osteonecrosis of the jaw in humans,[53] cow and sheep pigmentation,[54,55] and porcine hematological parameters.[56]

Supplementary Note 2

The Severe Morning Sickness phenotype refers to HG.

Six questions used to derive (classifications of) phenotypes:

1) Did you experience morning sickness during your pregnancy?
    A1: Yes
    A2: No
    A3: I'm not sure

2) Did you experience morning sickness during any of your pregnancies?
    A1: Yes
    A2: No
    A3: I'm not sure

3) How severe was your morning sickness?
    A1: Mild (occasional bouts of queasiness or nausea, did not require treatment)
    A2: Moderate (nausea and some vomiting, but did not require treatment)
    A3: Severe (Severe nausea and vomiting that required treatment)
    A4: Very severe (requiring hospitalization and intravenous fluid (IV) therapy)
    A5: I'm not sure

4) For the pregnancy in which you had the worst morning sickness, how severe was your morning sickness?
    A1: Mild (occasional bouts of queasiness or nausea, did not require treatment)
    A2: Moderate (nausea and some vomiting, but did not require treatment)
    A3: Severe (Severe nausea and vomiting that required treatment)
    A4: Very severe (requiring hospitilization and intravenous fluid (IV) therapy)
    A5: I'm not sure

5) Did you experience morning sickness?
    A1: Yes, it was mild enough that I did not require treatment
    A2: Yes, it was severe enough that I required treatment
    A3: No
    A4: I'm not sure

6) Did you ever get intravenous fluid (IV) therapy for nausea and vomiting (morning sickness) during pregnancy?
    A1: Yes
    A2: No
    A3: I don't know

From these questions, we mapped onto a phenotype that scales morning sickness severity as follows:

HG cases were defined as individuals who gave ANY of the following answers: Q3:A4, Q4:A4, or Q6:A1, and also did not give ANY of the following: Q1:A2, Q2:A2, or Q5:A3.

Controls were defined as individuals who gave ANY of the following answers: Q1:A2, Q2:A2, or Q5:A3, but also did not give other answers that were inconsistent.

Phenotypes for cases and controls were considered mutually exclusive and reasonable filters were applied to include only individuals whose responses were logically consistent.

HG cases (Q3:A4, Q4:A4, Q6:A1 and NOT Q1:A2, Q2:A2, or Q5:A3)
1,306 research participants

Controls (Q1:A2, Q2:A2, Q5:A3)
15,756 research participants

For the ordinal phenotype related to HG analyzed in SCAN2, data were pulled from the following four questions:

1) "Did you experience morning sickness during your pregnancy? (morning_sickness)" (Yes, No, I'm not sure)

2) "Did you experience morning sickness during any of your pregnancies? (morning_sickness_any)" (Yes, No, I'm not sure)

3) "How severe was your morning sickness? (morning_sickness_severity)"
1=Mild (occasional bouts of queasiness or nausea, did not require treatment),
2=Moderate (nausea and some vomiting, but did not require treatment),
3=Severe (Severe nausea and vomiting that required treatment),
4=Very severe (requiring hospitalization and intravenous fluid (IV) therapy), NA=I'm not sure

4) "For the pregnancy in which you had the worst morning sickness, how severe was your morning sickness?

(worst_morning_sickness_severity)"

(1=mild,2=moderate,3=severe,4=very_severe,NA=I'm not sure)

From these questions NVP level was defined on a five-point scale, from “none” to “very severe,” as follows: The phenotype was scored 0 on a five-point scale if a participant reported a negative answer to either of the first two questions (morning_sickness or morning_sickness_any). Scores 1 through 4 were defined from answers to questions 3 and 4 (morning_sickness_severity and worst_morning_ sickness_severity). When a participant answered both questions, the highest-scoring answer was used as the response. The phenotype was set to NA for the answer “I’m not sure” to any of the four questions.
