# Supplementary material for: Placenta and appetite genes GDF15 and IGFBP7 are associated with hyperemesis gravidarum
Source: Nat Commun. 2018 Mar 21;9:1178. doi: 10.1038/s41467-018-03258-0 (PMC5862842; doi:10.1038/s41467-018-03258-0)
Supplement: Supplementary file 2 — Description of Additional Supplementary Files(PDF 799 kb) [file 41467_2018_3258_MOESM2_ESM.pdf]

Description of Additional Supplementary Files

File Name: Supplementary Data 1
Description: Annotation using query SNPs rs45543339, rs143409503, and variants with r2 >= 0.2.

File Name: Supplementary Data 2
Description: SNPs associated with altered expression of gene GDF15
Legend: no legend.
